# Determining the Optimal (Neo)Adjuvant Regimen for Human Epidermal Growth Factor Receptor 2-Positive Breast Cancer Regarding Survival Outcome: A Network Meta-Analysis

**DOI:** 10.3389/fimmu.2022.919369

**Published:** 2022-06-30

**Authors:** Yu-Wen Cai, Zhi-Ming Shao, Ke-Da Yu

**Affiliations:** ^1^ Department of Breast Surgery, Fudan University Shanghai Cancer Center, Shanghai, China; ^2^ Shanghai Medical College, Fudan University, Shanghai, China; ^3^ Shanghai Key Laboratory of Breast Cancer, Shanghai, China

**Keywords:** breast cancer, (neo)adjuvant, HER2-positive, disease-free survival, network meta-analysis

## Abstract

**Background:**

The optimal (neo)adjuvant regimen for human epidermal growth factor receptor-2 (HER2)-positive breast cancer regarding survival outcomes remains unclear.

**Methods:**

We searched Web of Science, PubMed, and the Cochrane Central Register of Controlled Trials systematically to find out randomized controlled studies, up to January 2022, that compared different anti-HER2 regimens in the (neo)adjuvant setting. The primary endpoint was disease-free survival (DFS). We used a Bayesian statistical model to combine direct and indirect comparisons and used odds ratios (ORs) to pool effect sizes and performed the surface under the cumulative ranking area (SUCRA) curves to estimate the ranking probabilities of various regimens. For survival outcomes, we performed two parallel analyses, one based on data from both neoadjuvant and adjuvant studies and the other specific to adjuvant studies. All statistics were two-sided.

**Results:**

Fifteen studies were finally enrolled. Regarding DFS, the overall analysis indicated that the top two regimens for HER2-positive breast cancer were chemotherapy plus trastuzumab with lapatinib, and chemotherapy plus trastuzumab with pertuzumab (SUCAR of 81% and 79%, respectively), with the OR of 0.99 [95% confidence interval (CI), 0.59 to 1.54]; the parallel analysis specific to adjuvant trials indicated that the top two regimens were chemotherapy plus trastuzumab with sequential neratinib, and chemotherapy plus trastuzumab with pertuzumab (SUCRA of 80% and 76%, respectively), with the OR of 1.04 (95% CI, 0.63 to 1.73). The dual-target therapy that combines trastuzumab and pertuzumab showed the highest risk of inducing cardiac events, with an SUCRA of 92%.

**Conclusions:**

Chemotherapy plus trastuzumab and pertuzumab might be the optimal regimen for HER2-positive breast cancer in improving the survival rate. However, the cardiotoxicity of this dual-target therapy should be taken care of.

## Introduction

According to the 2020 global cancer statistics, female breast cancer has become the most commonly diagnosed cancer globally. It was estimated that there were 2.3 million new breast cancer cases in 2020 (1). Approximately 15% to 20% of breast cancer patients are human epidermal growth factor receptor 2 (HER2) positive (2), which are associated with high disease recurrence and poor prognosis (3). Since late 2006, trastuzumab, a HER2-targeted monoclonal antibody, has been the standard care for this breast cancer subtype, and this was based on the results from several landmark trials that demonstrated a significant association between chemotherapy plus trastuzumab and better overall survival (OS) and progression-free survival in HER2-positive breast cancer (4–6). However, it was reported that among patients with early-stage, HER2-positive breast cancer who received trastuzumab and adjuvant chemotherapy, there was still a recurrence rate of approximately 16%–22% (7, 8), and among patients with metastatic breast cancer, 22% to 25% of them displayed primary or secondary resistance to HER2-targeted therapies (9, 10). Therefore, the focus of research has now shifted to finding strategies that can overcome resistance to HER2-targeted therapies so as to further improve patient outcomes.

The currently available HER2-targeted agents that are approved by the US Food and Drug Administration for treating HER2-positive breast cancer mainly included the following categories: HER2-targeted monoclonal antibodies, such as trastuzumab and pertuzumab; tyrosine kinase inhibitors (TKIs), such as lapatinib, neratinib, and tucatinib; and antibody-drug conjugates, which include trastuzumab emtansine (T-DM1) and trastuzumab deruxtecan (11). These agents can be used either alone or in combination, which has created many treatment choices that prompted us to find out the optimal one for this disease. Two previously published network meta-analyses (NMA) on HER2-positive breast cancer tried to integrate efficacy and safety information of all neoadjuvant regimens tested in clinical trials by combining both direct and indirect evidence (12, 13). However, they mainly focused on the outcome of pathological complete response, while the more important survival information was not integrated yet. Therefore, focusing on the survival data from relevant clinical trials, we conducted the present NMA to provide an updated overview on the comparative efficacy of the currently available (neo)adjuvant regimens for HER2-positive breast cancer.

## Methods

### Search Strategy

The present study was reported according to the Preferred Reporting Items for Systematic Reviews incorporating Network Meta Analyses (PRISMA-NMA) statement (14). Web of Science, PubMed, and the Cochrane Central Register of Controlled Trials were searched systematically. Only English publications were selected, and the search algorithm was as follows: “(mammary OR breast) AND (tumor OR carcinoma OR cancer) AND (human epidermal growth factor receptor 2 OR HER2 OR ERBB2) AND (positive) AND (adjuvant OR neoadjuvant OR preoperative) AND (therapy OR regimen OR treatment).” References of relevant studies were also reviewed carefully to find other relevant trials. The last search was performed in January 2022.

### Selection Criteria

The inclusion criteria were as follows: (a) randomized controlled trials that focused on (neo)adjuvant therapy for HER2-positive breast cancer; (b) trials included at least two treatment arms [one of the single-use or different combinations of monoclonal antibodies, TKIs, antibody–drug conjugates, and/or chemotherapy were considered as one arm]; and (c) reported the hazard ratio (HR) and its 95% confidence interval (CI) of survival outcomes. Studies that only recruited elderly patients (over 65 years old) were excluded. To reduce bias, studies that only reported the HR of partial participants (such as those who achieved pathological complete response) were also excluded. If multiple reports were available for the same trial, only the latest version that reported the corresponding survival data was enrolled.

### Outcomes

The primary endpoint of the present study was DFS, which was defined as the time from randomization to recurrence of invasive breast cancer at local, regional, or distant sites; contralateral invasive breast cancer; second non-breast malignancy; or death as a result of any cause, whichever occurred first. If not reported in a certain study, DFS would be substituted by event-free survival, progression-free survival, invasive disease-free survival, or distant DFS in order. Additionally, if any of the above candidate outcomes were reported as the primary outcome, it would be selected with priority. Secondary endpoints included OS, defined as the time from randomization to death as a result of any cause, and safety outcomes. Small differences in the definitions among enrolled studies were allowed.

### Data Collection and Bias Assessment

Data from the enrolled studies were extracted by two authors independently. The following information was collected: first author’s name, year of publication, trial name, the phase of the study, treatment settings, sample size, treatment arms and their corresponding number of patients, hormone receptor status, treatment duration, follow-up time, primary outcome, and survival outcomes reported with HR with 95% CI. The numbers of grade 3 or 4 adverse events according to the National Cancer Institute Common Terminology Criteria, version 3.0, together with the number of patients treated were also collected. Other grading standards were substituted if not reported. Only the intention-to-treat data were collected. Also, for safety outcomes, only those that were reported by three or more studies and could form a closed loop would be analyzed and reported. The quality of enrolled studies was assessed by version 2 of the Cochrane risk-of-bias tool for randomized trials (RoB 2), which contains five domains for evaluating the risk of bias in randomized trials: randomization process, deviations from intended interventions, missing outcome data, measurement of the outcome, and selection of the reported result (15). Disputes were resolved through discussion, or a third author would join to make a decision.

### Statistical Analysis

Firstly, data (HR with 95% CI for survival outcomes and the total number of patients treated in each arm, together with the corresponding incidence of events for safety outcomes) comparing the same treatment arms in terms of the same outcomes were integrated using traditional meta-analyses. By recalling JAGS in R for Markov chain Monte Carlo (MCMC) sampling, the “gemtc” R package was then used to perform NMA, in a Bayesian random-effects model, and the odds ratios (ORs) for survival and safety outcomes were used to pool effect sizes. A total of 200,000 simulations were generated for each of the sets of different initial values, and the first 5,000 simulations, as an annealing process, were discarded. Brooks-Gelman-Rubin diagnostic and trace plots were then performed to check the convergence of the model (16).

Other than the overall analysis that combined both the neoadjuvant and adjuvant data, a parallel analysis specific to adjuvant data was also performed to reduce bias. The overall analysis was based on more comprehensive and longer (including both the neoadjuvant and adjuvant periods) data compared with the parallel analysis. However, the primary endpoint of most neoadjuvant studies is pathological complete response, which might have caused some bias regarding survival data. In contrast, the parallel analysis specific to adjuvant studies, the primary endpoint of which is mostly survival outcomes, was based on more accurate survival data. Therefore, it is necessary to perform the two analyses simultaneously, and only therapies that pass the test of the two analyses would be considered to be truly effective.

Inconsistency tests were performed using the node-splitting method by separating the direct and indirect evidence of the same comparison (17). *I^2^* tests were performed to assess the heterogeneity, with *I^2^* > 50% indicating significant heterogeneity. Additionally, sensitivity analyses were also performed by omitting each article sequentially to test the robustness of the primary results. At last, the ranking probabilities of treatment arms in terms of different outcomes were estimated using the surface under the cumulative ranking area (SUCRA) (18). R software (version 4.1.1) and STATA (version 15.0, Stata MP) were used to perform the above statistical analyses and generate plots, with *P* value less than 0.05 considered statistically significant.

## Results

### Study Selection

**Figure 1:**
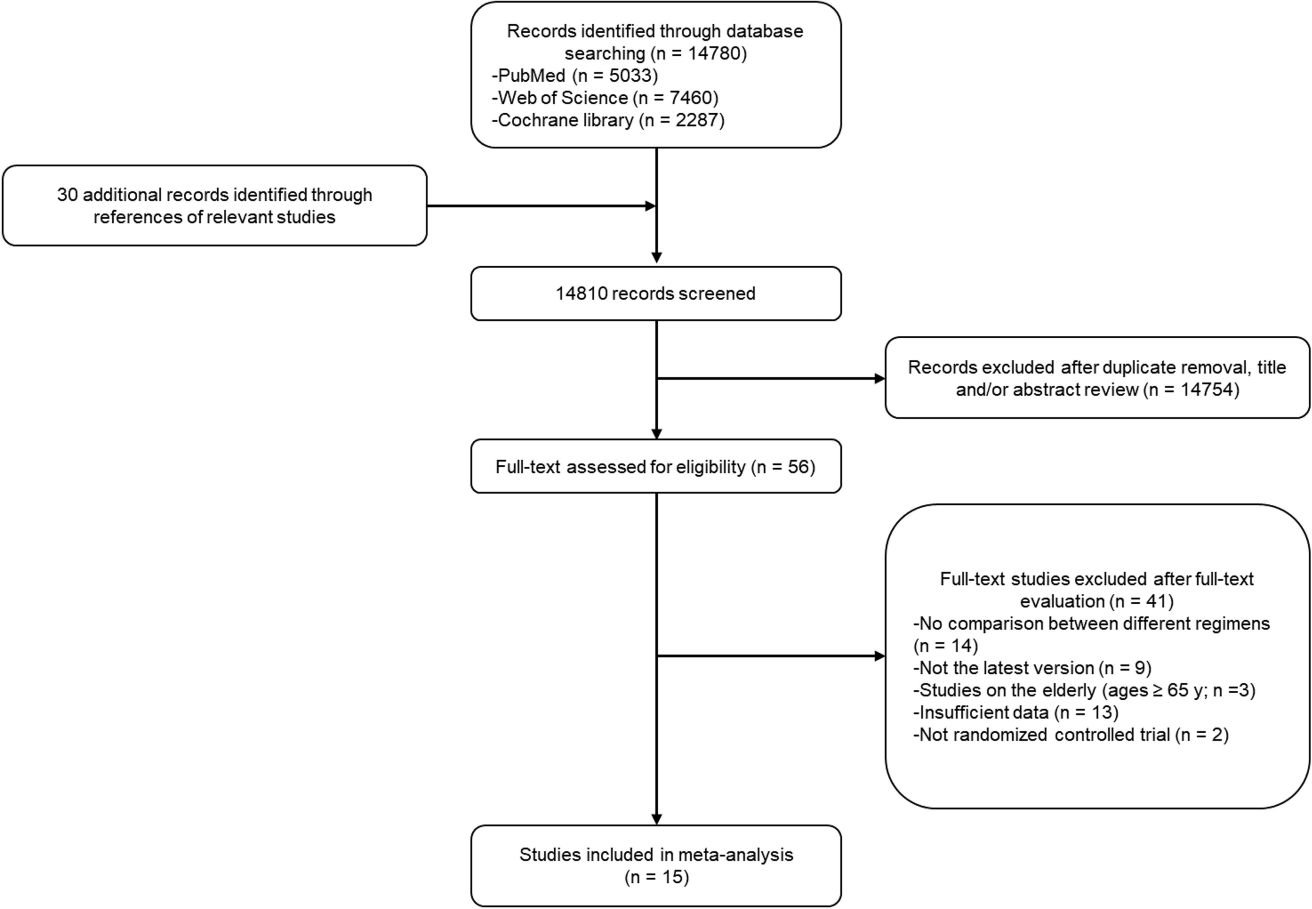
Flowchart of the selection process of studies to be enrolled.

A total of 14,780 potentially relevant studies in the databases and 30 additional studies from references were filtered, from which only 15 studies were finally enrolled in the present analysis (7, 8, 19–31). The detailed selection process is shown in **Figure 1**. The enrolled studies were published from 2009 to 2021, and the sample size ranged from 232 to 8,381, with a total sample size of 33,226 in the overall analysis. Five of the enrolled studies were of neoadjuvant setting, while the remaining 10 studies were of adjuvant setting. The baseline characteristics of the enrolled studies are summarized in **Table 1**. The anti-HER2 duration of the majority of enrolled studies was 1 year, except that of the FinHER Trial (25) was 9 weeks. In general, the quality of enrolled studies was medium to high, as shown in **Supplementary Material 1**.

**Table 1:** Baseline characteristic of enrolled studies.

Study *^a^*	Treatment arms	Neoadjuvant therapy *^c^*	Adjuvant therapy	Sample size	HoR+, n (%) *^d^*	Primary endpoint	Survival *^f^*	HR (95% CI) for DFS *^g^*	HR (95% CI) for OS
NeoSphere (N = 417, phase II). 60 ms; 1 y (19). *^b^*	CT + Tzmb	D + Tzmb	Tzmb + FEC	107	50 (46.7)	pCR	PFS	Ref.	NR
	CT + Tzmb + Pzmb	D + Tzmb + Pzmb	Tzmb + FEC	107	50 (46.7)			0.69 (0.34–1.40)	NR
	Tzmb + Pzmb	Tzmb + Pzmb	Tzmb + (D → FEC)	107	51 (48.1)			1.25 (0.68–2.30)	NR
	CT + Pzmb	D + Pzmb	Tzmb + FEC	96	46 (47.9)			2.05 (1.07–3.93)	NR
NeoALTTO (N = 455, phase III). 3.84 ys; 52 wks (20). *^b^*	CT + Lapa	P + Lapa	FEC → Lapa	154	80 (51.9)	pCR	EFS, OS	1.06 (0.66–1.96)	0.86 (0.45–1.63)
	CT + Tzmb	P + Tzmb	FEC → Tzmb	149	75 (50.3)			Ref.	Ref.
	CT + Tzmb + Lapa	P + Tzmb + Lapa	FEC → (Tzmb + Lapa)	152	77 (50.7)			0.78 (0.47–1.28)	0.62 (0.30–1.25)
NOAH (N = 235, phase III). 5.4 ys; 52 wks (21). *^b^*	CT	AP → P → CMF	–	118	42 (35.6)	EFS	EFS, OS	Ref.	Ref.
	CT + Tzmb	(AP → P → CMF) + Tzmb	Tzmb	117	42 (35.9)			0.64 (0.44–0.93)	0.66 (0.43–1.01)
TEACH (N = 3,147, phase III). 48 ms; 1 y (22).	CT + Lapa	–	(A/E ± T) + Lapa	1,571	932 (59.3)	DFS	DFS, OS	0.83 (0.70–1.00)	0.99 (0.74–1.31)
	CT	–	A/E ± T	1,576	927 (58.8)			Ref.	Ref.
ExteNET (N = 2,840, phase III). 5.2 ys; 1 y (23).	CT + Tzmb → Nera	–	(A/E ± T) + Tzmb → Nera	1,420	816 (57.5)	iDFS	iDFS, OS	0.73 (0.57–0.92)	0.95 (0.75–1.21)
	CT + Tzmb	–	(A/E ± T) + Tzmb	1,420	815 (57.4)			Ref.	Ref.
NSABP B-31 and N9831. (N = 4,046, phase III). 8.4 ys; 1 y (8).	CT	–	AC → P	2,018	1,105 (54.8)	DFS	DFS, OS	Ref.	Ref.
	CT + Tzmb	–	(AC → P) + Tzmb	2,028	1,110 (54.7)			0.60 (0.53–0.68)	0.63 (0.54–0.73)
ALTTO (N = 8381, phase III). 4.5 ys; 1 y (24).	CT + Tzmb + Lapa	–	CT + Tzmb + Lapa	2,093	1,203 (57.5)	DFS	DFS, OS	0.84 (0.70–1.02)	0.80 (0.62–1.03)
	CT + Tzmb → Lapa	–	CT + Tzmb → Lapa	2,091	1,205 (57.6)			0.96 (0.80–1.15)	0.91 (0.71–1.16)
	CT + Lapa	–	CT + Lapa	2,100	1,197 (57.0)			1.34 (1.13–1.60)	1.36 (1.09–1.72)
	CT + Tzmb	–	CT + Tzmb	2,097	1,200 (57.2)			Ref.	Ref.
FinHer (N = 232, phase III). 62 ms; 9 wks (25).	CT + Tzmb	–	D/V → FEC → Tzmb	116	58 (50.0)	DDFS	DDFS, OS	0.65 (0.38–1.12)	0.55 (0.27–1.11)
	CT	–	D/V → FEC	116	51 (44.0)			Ref.	Ref.
HERA (N = 3,399, phase III). 11 ys; 1 y (7).	CT + Tzmb	–	(A/E ± T) + Tzmb	1,702	859 (50.5)	DFS	DFS, OS	0.76 (0.68–0.86)	0.74 (0.64–0.86)
	CT	–	A/E ± T	1,697	855 (50.4)			Ref.	Ref.
FNCLCC-PACS 04 (N = 528, phase III). 115 ms; 1 y (26).	CT + Tzmb	–	(FEC or ED) → Tzmb	260	151 (58.1)	DFS	DFS, OS	0.77 (0.57–1.03)	0.82 (0.56–1.21)
	CT	–	FEC or ED	268	164 (61.2)			Ref.	Ref.
BCIRG-006 (N = 2147, phase III). 10.3 ys; 1 y (27).	CT + Tzmb	–	(AC → D) + Tzmb	1,074	578 (53.8)	DFS	DFS, OS	0.70 (0.60–0.83)	0.64 (0.52–0.79)
	CT	–	AC → D	1,073	576 (53.7)			Ref.	Ref.
KRISTINE (N = 444, phase III). 37 ms; 54 wks (28). *^b^*	T-DM1 + Pzmb	T-DM1 + Pzmb	T-DM1 + Pzmb	223	139 (62.3)	pCR	EFS, OS	2.61 (1.36–4.98)	1.21 (0.37–3.96)
	CT + Tzmb + Pzmb	DB + Tzmb + Pzmb	Tzmb + Pzmb	221	137 (62.0)			Ref.	Ref.
KAITLIN (N = 1,846, phase III). 57 ms; 1 y (29).	CT + T-DM1 + Pzmb	–	A/E → (T-DM1 + Pzmb)	928	519 (55.9)	iDFS	iDFS, OS	0.98 (0.72–1.32)	1.40 (0.89–2.21)
	CT + Tzmb + Pzmb	–	A/E → (T + Tzmb + Pzmb)	918	516 (56.2)			Ref.	Ref.
CALGB 40601 (N = 305, phase III). 83 ms; 1 y (30). *^b^*	CT + Tzmb + Lapa	P + Tzmb + Lapa	AC + Tzmb	118	70 (59.3)	pCR	RFS, OS	0.32 (0.14–0.71)	0.34 (0.12–0.94)
	CT + Tzmb	P + Tzmb	AC + Tzmb	120	70 (58.3)			Ref.	Ref.
	CT + Lapa	P + Lapa	AC + Tzmb	67	39 (58.2)			1.50 (0.82–2.77)	1.17 (0.51–2.71)
APHINITY (N = 4,804, phase III). 74 ms; 1 y (31).	CT + Tzmb + Pzmb	–	Tzmb + Pzmb → (A/E-T or DB)	2,400	1,536 (64.0)	iDFS	iDFS, OS	0.76 (0.64–0.91)	0.85 (0.67–1.07)
	CT + Tzmb	–	Tzmb → (A/E-T or DB)	2,404	1,546 (64.3)			Ref.	Ref.

A, doxorubicin; B, carboplatin; C, cyclophosphamide; CI, confidence interval; CT, chemotherapy; D, docetaxel; DDFS, distant disease-free survival; DFS, disease-free survival; E, epirubicin; EFS, event-free survival; F, fluorouracil; mFU, median follow-up; HER2, human epidermal growth factor receptor-2; HoR, hormone receptor; HR, hazard ratio; iDFS, invasive disease-free survival; Lapa, lapatinib; M, methotrexate; ms, months; Nera, neratinib; NR, not report; OS, overall survival; tzmb, trastuzumab; P, paclitaxel; pCR, pathological complete response; PFS, progression-free survival; Pzmb, pertuzumab; RFS, recurrence-free survival; T, taxane; T-DM1, trastuzumab emtansine; Tzmb, trastuzumab; V, vinorelbine; y(s), year(s).

a Listed are trial name, median follow-up time, and the longest anti-HER2 duration.

b Studies of neoadjuvant design.

c For studies of only adjuvant design, a small proportion of participants received neoadjuvant chemotherapy are allowed.

d If the hormone receptor status was not available, the estrogen receptor status was listed.

f Reported are survival data that were collected and analyzed.

g DFS, if not reported in one study, would be substituted by EFS, PFS, iDFS, DDFS, or RFS (in order). Additionally, if any of the above outcomes was reported as primary outcome, it would be selected with priority.

### DFS Network

**Figure 2:**
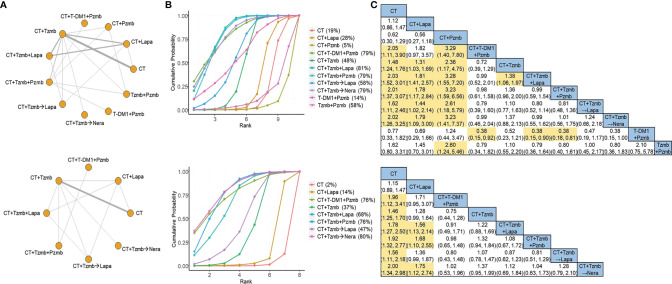
Network meta-analysis for disease-free survival. **(A)** Network plots for disease-free survival in the overall analysis (top panel) and in the parallel analysis (bottom panel). **(B)** Ranking probabilities of surface under the cumulative ranking curve for disease-free survival in the overall analysis (top panel) and in the parallel analysis (bottom panel). **(C)** Cross-comparison odds ratios and their corresponding 95% confidence intervals for disease-free survival in the overall analysis (top panel) and in the parallel analysis (bottom panel). CT, chemotherapy; Lapa, lapatinib; Nera, neratinib; Pzmb, pertuzumab; T-DM1, trastuzumab emtansine; Tzmb, trastuzumab.

In the overall analysis, a total of 15 studies (7, 8, 19–31) with 11 treatment arms were involved in the NMA of DFS, as shown in **Figure 2A** (top panel). According to the SUCRA estimates, the top two regimens for HER2-positive breast cancer were trastuzumab plus lapatinib with chemotherapy and trastuzumab plus pertuzumab with chemotherapy [SUCRA of 81% and 79%, respectively; **Figure 2B** (top panel)], with the cross-comparison OR of 0.99 [95% CI: 0.59 to 1.54; **Figure 2C** (top panel)]. Of note, there was significant heterogeneity in the traditional pair-wise comparison of trastuzumab plus lapatinib with chemotherapy versus lapatinib plus chemotherapy (*I* ^2^ = 85.5%), as shown in **Supplementary Material 2**. No significant inconsistency was observed between direct and indirect evidence, as shown in **Supplementary Material 3**. A total of 10 studies (7, 8, 22–27, 29, 31) with eight treatment arms were involved in the parallel analysis specific to adjuvant studies, as illustrated in **Figure 2A** (bottom panel). According to the SUCRA estimates, the top two therapies were trastuzumab with sequential neratinib based on chemotherapy and trastuzumab plus pertuzumab with chemotherapy [SUCRA of 80% and 76%, respectively; **Figure 2B** (bottom panel)], with the OR of the latter regimen compared with the former of 1.04 [95% CI: 0.63 to 1.73; **Figure 2C** (bottom panel)].

### OS network

**Figure 3:**
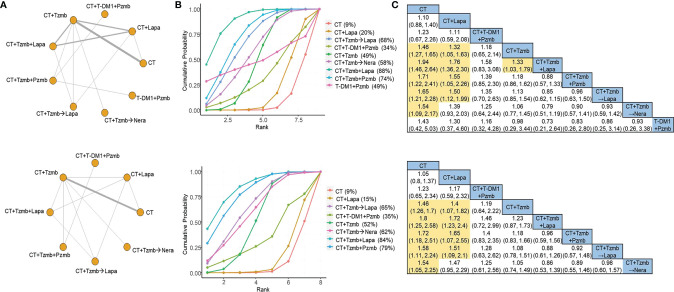
Network meta-analysis for overall survival. **(A)** Network plots for overall survival in the overall analysis (top panel) and in the parallel analysis (bottom panel). **(B)** Ranking probabilities of surface under the cumulative ranking curve for overall survival in the overall analysis (top panel) and in the parallel analysis (bottom panel). **(C)** Cross-comparison odds ratios and their corresponding 95% confidence intervals for overall survival in the overall analysis (top panel) and in the parallel analysis (bottom panel). CT, chemotherapy; Lapa, lapatinib; Nera, neratinib; Pzmb, pertuzumab; T-DM1, trastuzumab emtansine; Tzmb, trastuzumab.

For OS outcome, a total of 14 studies (7, 8, 20–31) were involved in the overall analysis and 10 studies (7, 8, 22–27, 29, 31) in the parallel analysis, as shown in **Figure 3A** (top panel) and **Figure 3A** (bottom panel), respectively. Both of the two analyses indicated that the top two therapies were trastuzumab plus lapatinib with chemotherapy and trastuzumab plus pertuzumab with chemotherapy [SUCRA of 88% and 74%, respectively, in the overall analysis (**Figure 3B**, top panel) and 84% and 79%, respectively, in the parallel analysis (**Figure 3B**, bottom panel)]. The cross-comparison OR was 0.88 (95% CI: 0.57 to 1.33) in the overall analysis (**Figure 3C**, top panel) and 0.96 (95% CI: 0.59 to 1.56) in the parallel analysis (**Figure 3C**, bottom panel), respectively. No significant heterogeneity was observed in the traditional pair-wise comparisons of OS, and the direct and indirect evidence was generally consistent, as shown in **Supplementary Materials 2**, **3**, respectively.

### Safety Network

The safety data of cardiac events, neutropenia, febrile neutropenia, diarrhea, vomiting, rash or erythema, hepatobiliary disorders, arthralgia, and fatigue were collected and analyzed in the study. The network and SUCRA plots for the above safety outcomes are shown in **Supplementary Materials 4**, **5**, respectively. The combination of trastuzumab and pertuzumab with or without chemotherapy showed the highest risk of inducing cardiac events, with a SUCRA of 92%. Trastuzumab with sequential neratinib based on chemotherapy had the highest risk of causing diarrhea, vomiting, rash or erythema, and fatigue, with the SUCRA of 94%, 92%, 98%, and 94%, respectively, while chemotherapy plus lapatinib had the highest risk of inducing neutropenia and hepatobiliary, with the SUCRA of 90% and 82%, respectively. Pertuzumab plus chemotherapy was most likely to cause febrile neutropenia, while chemotherapy alone was most likely to cause arthralgia, with the SUCRA of 79% and 74%, respectively.

### Sensitivity Analysis

Sensitivity analyses were performed by omitting a single study sequentially, and the results were generally consistent with the primary results (data not shown). Of note, the heterogeneity observed in the comparison of DFS of trastuzumab plus lapatinib with chemotherapy versus lapatinib plus chemotherapy became non-significant when the CALGB 40601 Trial (30) was omitted.

## Discussion

With the increase of anti-HER2 therapies and related head-to-head studies, it is vital to perform a comprehensive analysis to integrate relevant data. The previous NMA on HER2-positive breast cancer focused on either only neoadjuvant regimens using pathologic complete response as primary outcome (13, 32) or only adjuvant regimens using OS as primary outcome (33). In our study, however, more recent and complete studies were enrolled to compare regimens in both the neoadjuvant and adjuvant settings, and DFS was set as the primary efficacy indicator, which enhances the reliability of our results.

In the overall analysis that combined both neoadjuvant and adjuvant studies, our ranking results indicated that in terms of inducing DFS, the top two therapies were, in order, the combination of trastuzumab and TKI, and the dual-target anti-HER2 therapy that combines trastuzumab and pertuzumab, both on the basis of chemotherapy. For the neoadjuvant studies, although they had the advantages of longer treatment duration and more comprehensive data (including both the neoadjuvant and adjuvant periods), its primary endpoint was often pathological complete response rather than survival outcomes, which might have caused some bias regarding survival outcomes. Therefore, a parallel analysis that was specific to adjuvant studies was further performed. As a result, the top two regimens for HER2-positive breast cancer in terms of inducing DFS were trastuzumab with sequential lapatinib and the combination of trastuzumab and pertuzumab, both on the basis of chemotherapy. The combination of trastuzumab and TKI showed good efficacy only in the overall survival, and trastuzumab with sequential lapatinib with chemotherapy showed good efficacy only in the parallel analysis. Both of the above two therapies failed to pass the test of both analyses simultaneously, while only trastuzumab plus pertuzumab with chemotherapy succeeded. Therefore, we tended to infer that chemotherapy with dual-target anti-HER2 therapy that combines trastuzumab and pertuzumab was the optimal therapy for HER2-positive early breast cancer. This finding was consistent with the results of a previous NMA, in which therapies containing trastuzumab and pertuzumab were found to be most likely to be the best therapy in terms of achieving pathological complete response (13).

The sensitivity analyses indicated that the observed heterogeneity was mainly induced by the CALGB 40601 Trial. A potential reason might be the long follow-up time of CALGB 40601 Trial (nearly 7 years) (30) than that of the other two trials (less than 5 years) (20, 24). Nevertheless, there are still some limitations of our study. First, although the present NMA mainly focused on survival data, the primary outcome of some enrolled studies was not survival outcome, which, to a certain extent, reduced the statistical efficiency. Second, the definitions of outcomes, the dose and duration of therapies, the baseline chemotherapy regimens, and the follow-up time in the enrolled studies were not completely consistent, which might be a partial source of heterogeneity. Third, due to the limited data, we could not make further subgroup analysis regarding important clinical factors, such as hormone receptor status. Fourth, a total of 11 regimens were compared, but only 15 studies were enrolled; therefore, the majority of pair-wise comparisons was composed of only one or two studies. Fifth, we did not register the NMA prospectively. All the above limitations should be considered with caution when extrapolating our findings.

Collectively, the results suggested that chemotherapy plus dual-target anti-HER2 therapy that combines trastuzumab and pertuzumab was the optimal regimen for HER2-positive breast cancer. The cardiotoxicity of this dual-target therapy should be taken care of, and the cardiac function of patients receiving this therapy is recommended to be regularly reviewed. Studies of a larger scale involving more participants still need to validate our results further.

## Data Availability Statement

The original contributions presented in the study are included in the article/**Supplementary Material**. Further inquiries can be directed to the corresponding author.

## Author Contributions

Conception and design: K-DY; administrative support: K-DY and Z-MS; provision of study materials or patients: K-DY; collection and assembly of data: Y-WC and K-DY; data analysis and interpretation: Y-WC; manuscript writing: Y-WC and K-DY; final approval of manuscript: all authors. All authors contributed to the article and approved the submitted version.

## Conflict of Interest

The authors declare that the research was conducted in the absence of any commercial or financial relationships that could be construed as a potential conflict of interest.

## Publisher’s Note

All claims expressed in this article are solely those of the authors and do not necessarily represent those of their affiliated organizations, or those of the publisher, the editors and the reviewers. Any product that may be evaluated in this article, or claim that may be made by its manufacturer, is not guaranteed or endorsed by the publisher.
