# Supplementary material for: Determining the Optimal (Neo)Adjuvant Regimen for Human Epidermal Growth Factor Receptor 2-Positive Breast Cancer Regarding Survival Outcome: A Network Meta-Analysis
Source: Front Immunol. 2022 Jun 30;13:919369. doi: 10.3389/fimmu.2022.919369 (PMC9279606; doi:10.3389/fimmu.2022.919369)
Supplement: Supplementary file 1 [file DataSheet_1.zip › Supplementary Materials/Supplementary Material 1.pptx]

## Slide 1
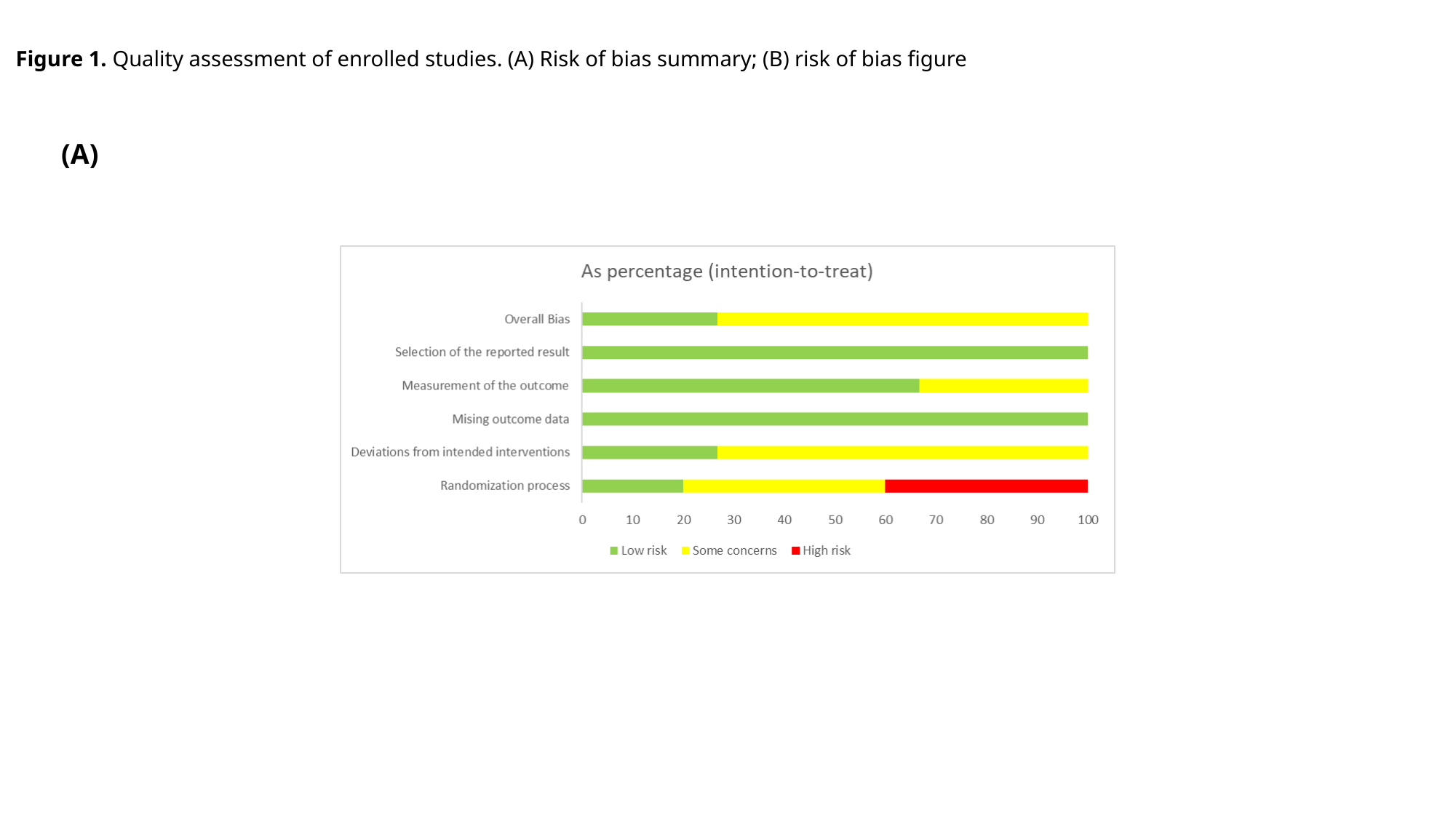

Figure 1. Quality assessment of enrolled studies. (A) Risk of bias summary; (B) risk of bias figure
(A)

## Slide 2
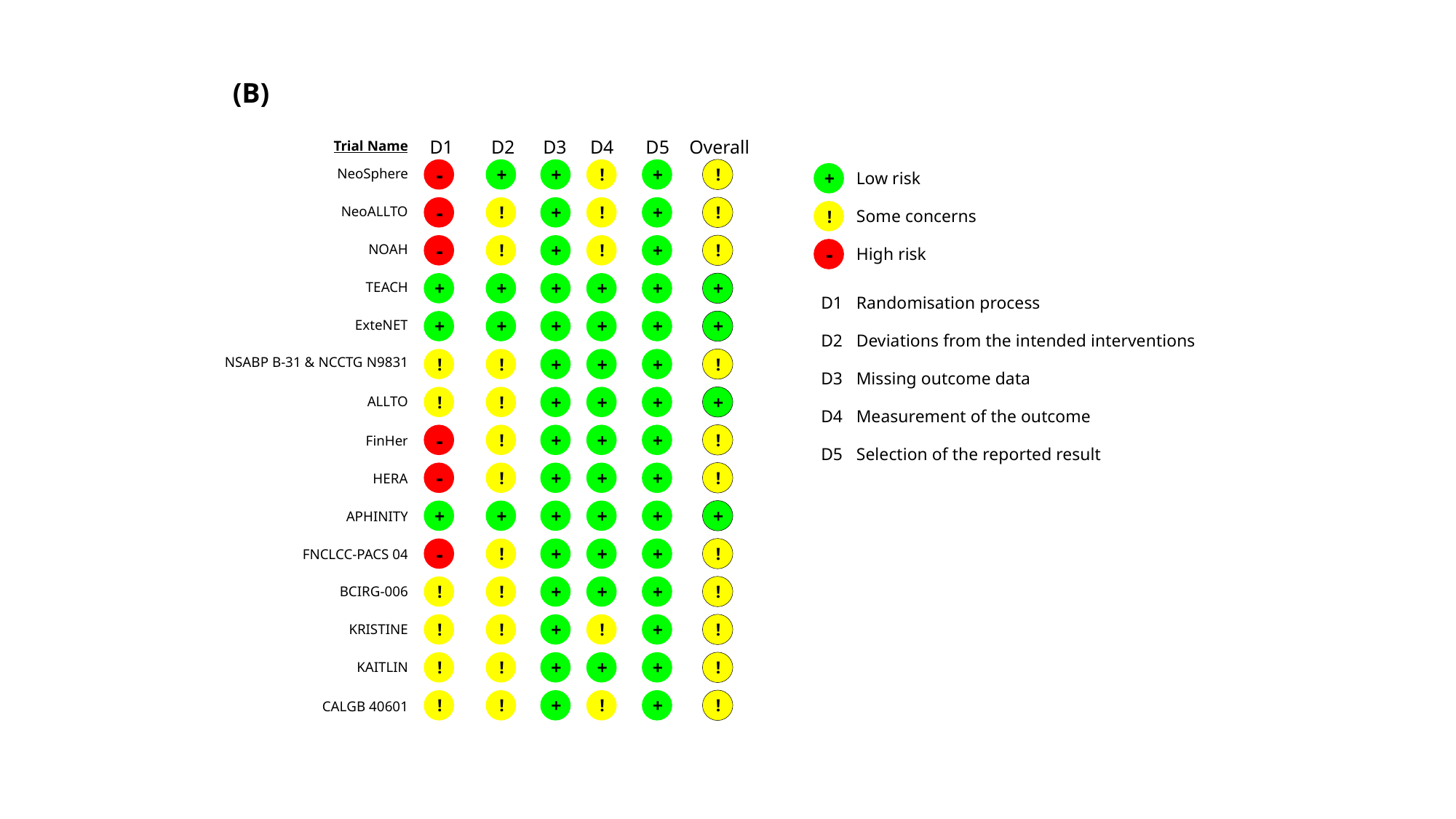

(B)
| Trial Name |
| --- |
| NeoSphere |
| NeoALLTO |
| NOAH |
| TEACH |
| ExteNET |
| NSABP B-31 & NCCTG N9831 |
| ALLTO |
| FinHer |
| HERA |
| APHINITY |
| FNCLCC-PACS 04 |
| BCIRG-006 |
| KRISTINE |
| KAITLIN |
| CALGB 40601 |
Overall
D4
D1
D2
D3
D5
-
+
+
!
+
!
| | Low risk |
| --- | --- |
| | Some concerns |
| | High risk |
+
-
!
+
!
+
!
!
-
!
+
!
+
!
-
+
+
+
+
+
+
| D1 | Randomisation process |
| --- | --- |
| D2 | Deviations from the intended interventions |
| D3 | Missing outcome data |
| D4 | Measurement of the outcome |
| D5 | Selection of the reported result |
+
+
+
+
+
+
!
!
+
+
+
!
!
!
+
+
+
+
-
!
+
+
+
!
-
!
+
+
+
!
+
+
+
+
+
+
-
!
+
+
+
!
!
!
+
+
+
!
!
!
+
!
+
!
!
!
+
+
+
!
!
!
+
!
+
!
