# Supplementary material for: Determining the Optimal (Neo)Adjuvant Regimen for Human Epidermal Growth Factor Receptor 2-Positive Breast Cancer Regarding Survival Outcome: A Network Meta-Analysis
Source: Front Immunol. 2022 Jun 30;13:919369. doi: 10.3389/fimmu.2022.919369 (PMC9279606; doi:10.3389/fimmu.2022.919369)
Supplement: Supplementary file 1 [file DataSheet_1.zip › Supplementary Materials/Supplementary Material 2.pptx]

## Slide 1
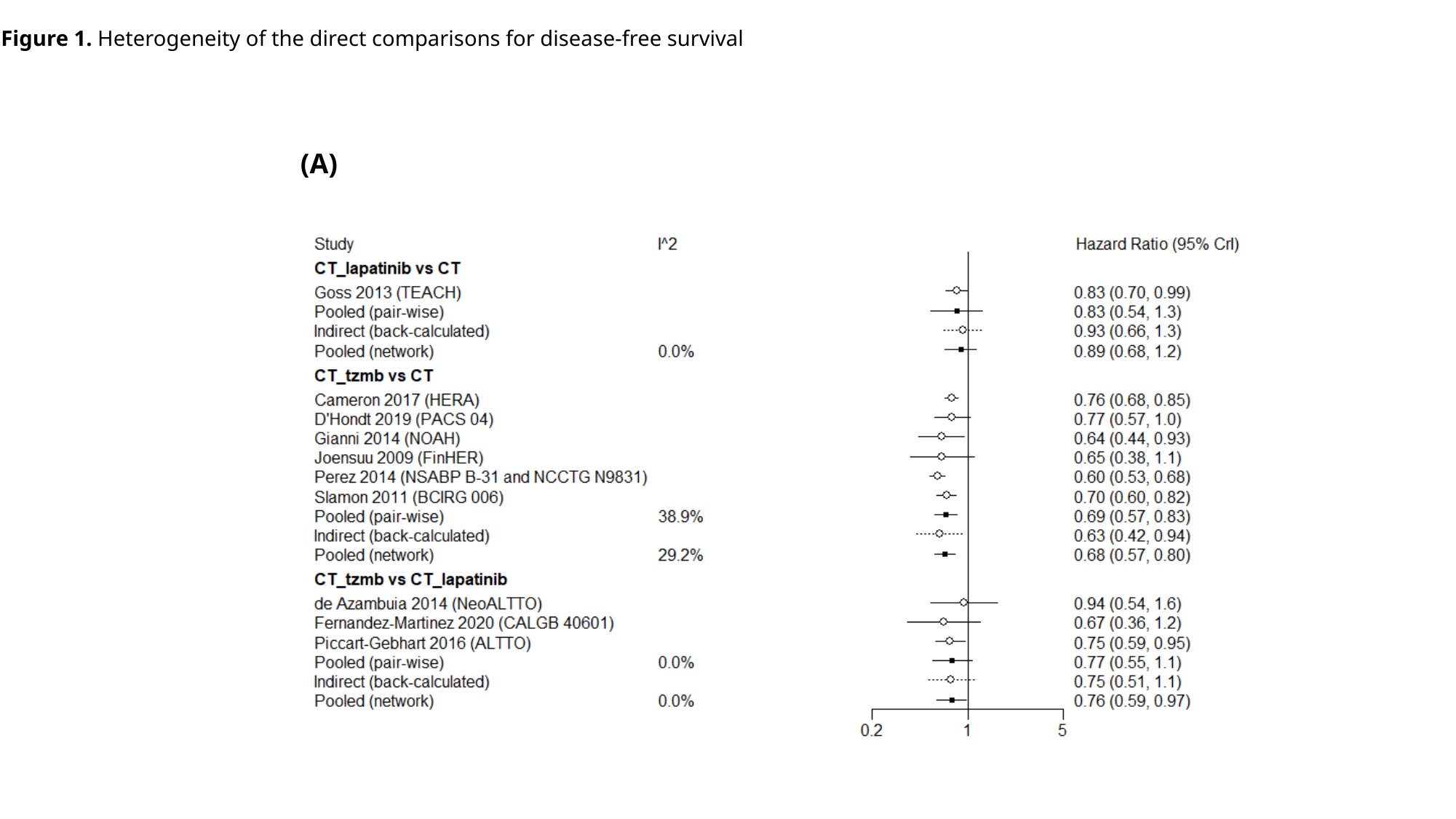

Figure 1. Heterogeneity of the direct comparisons for disease-free survival
(A)

## Slide 2
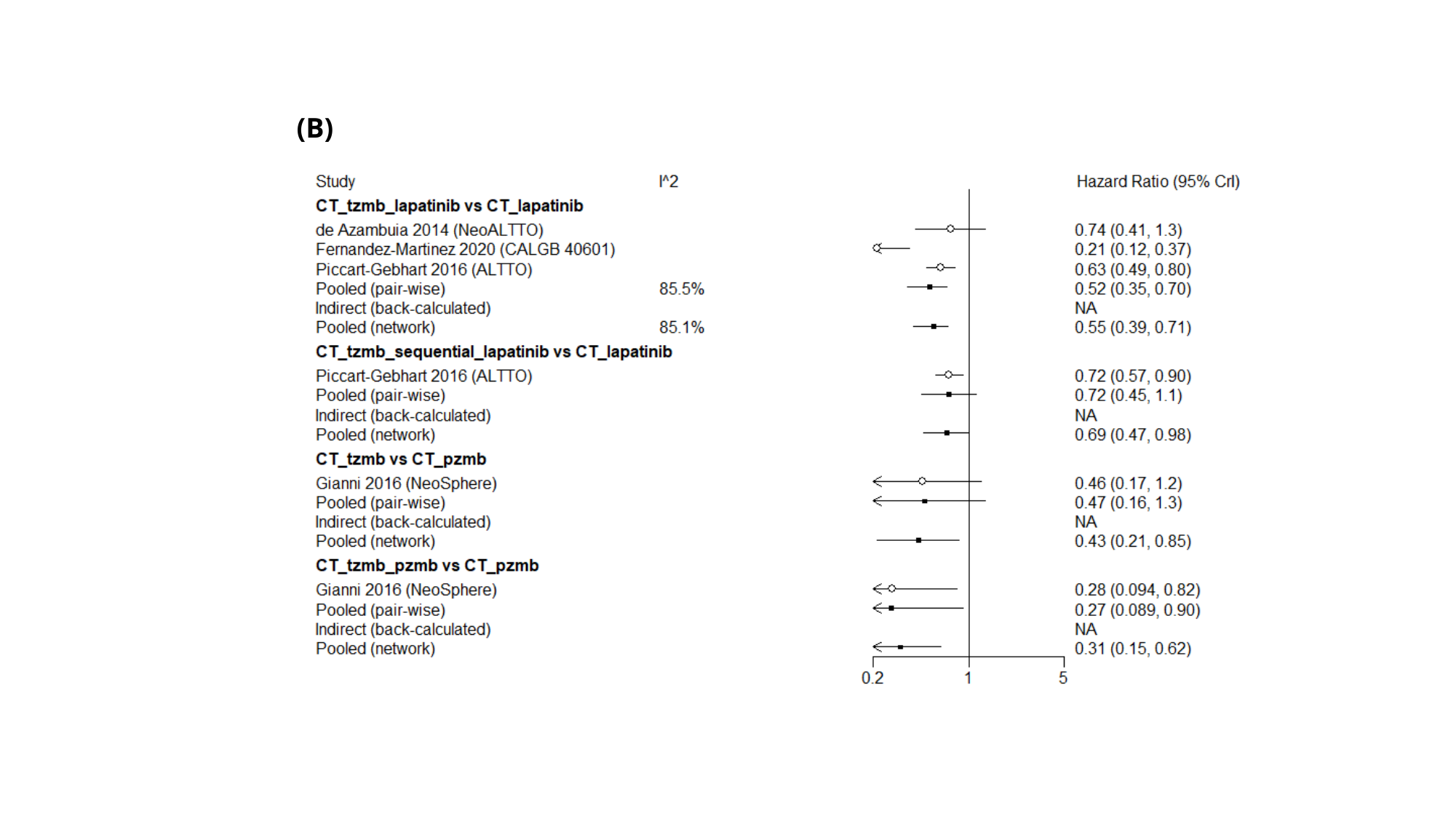

(B)

## Slide 3
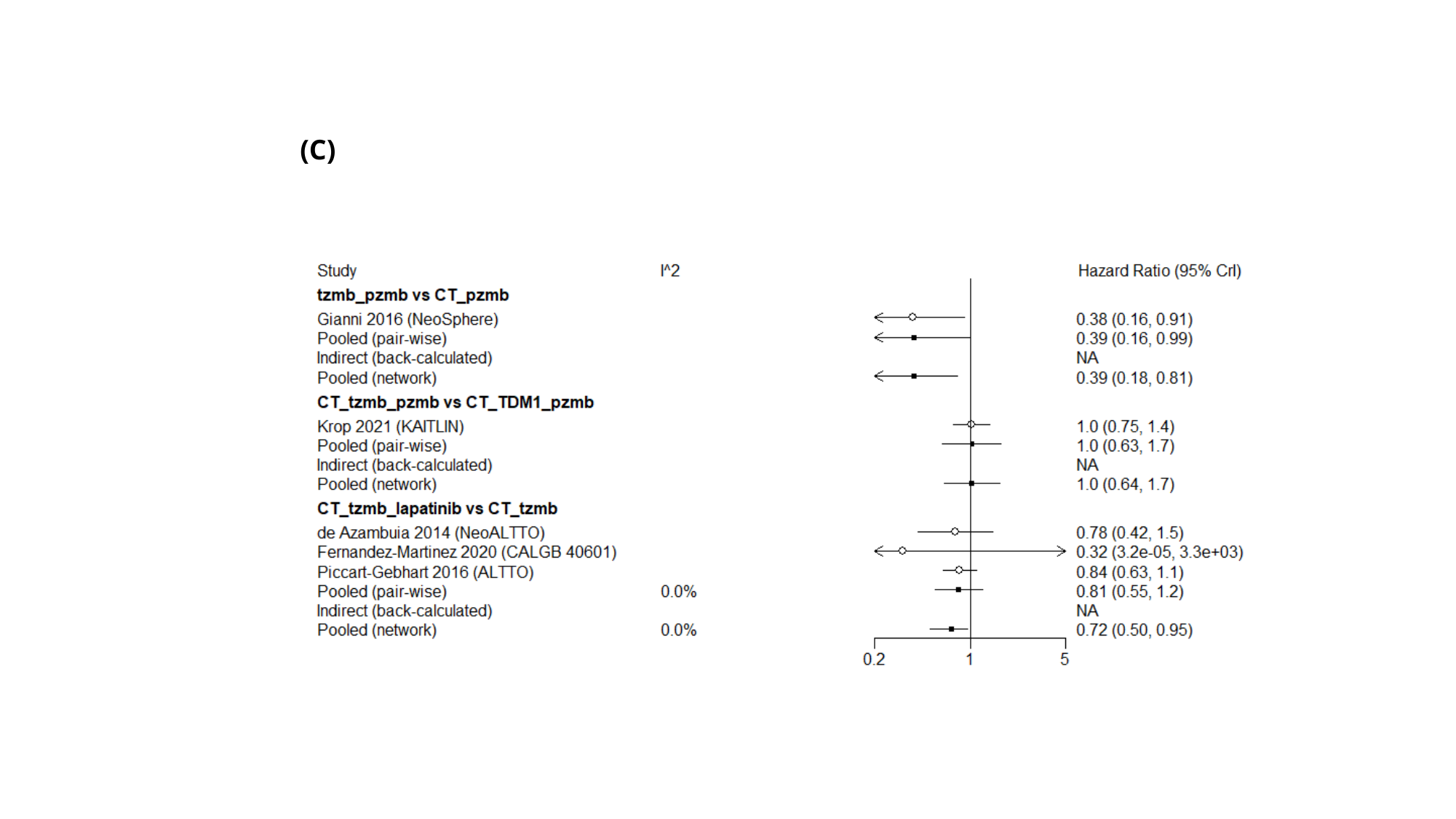

(C)

## Slide 4
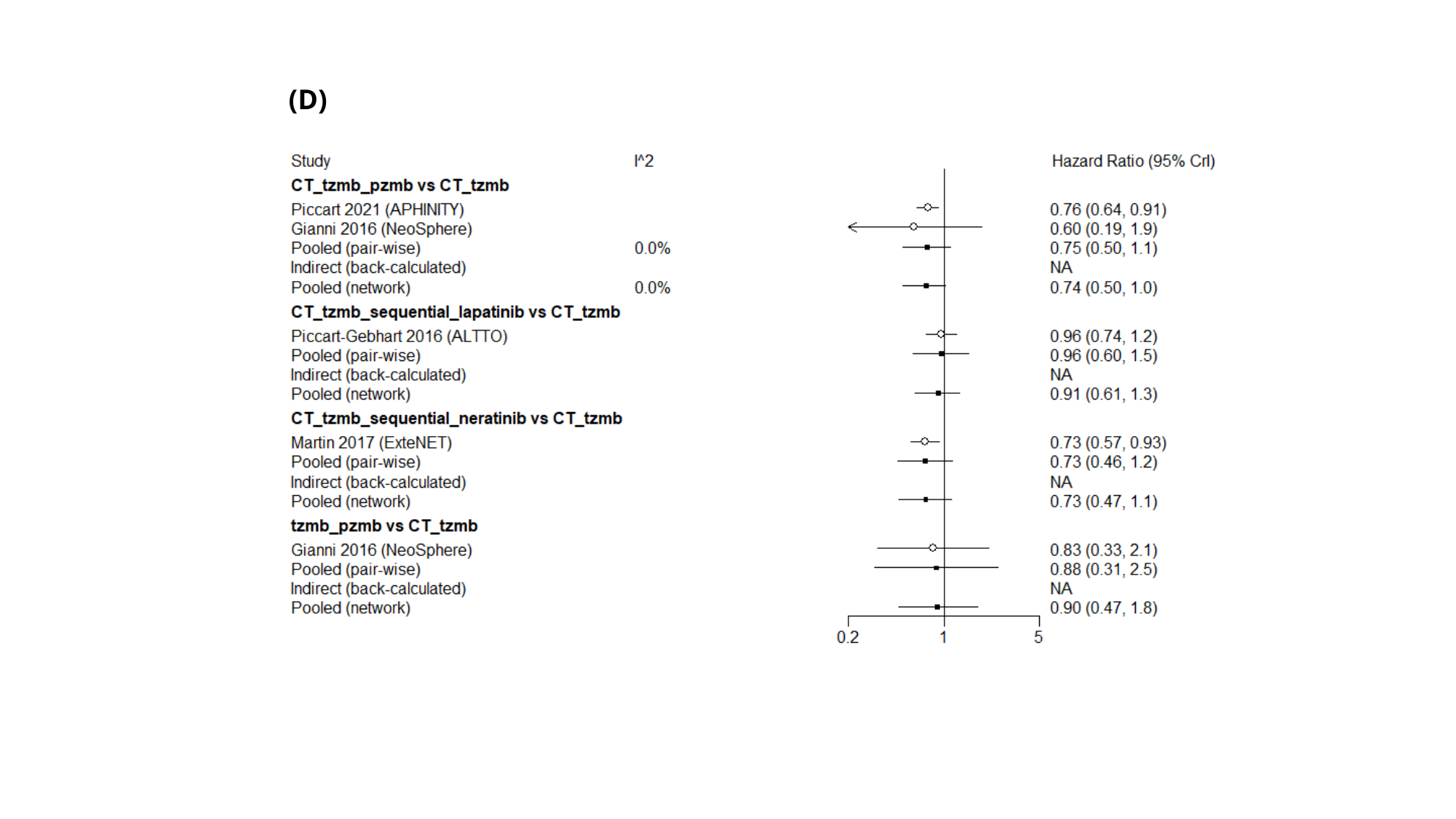

(D)

## Slide 5
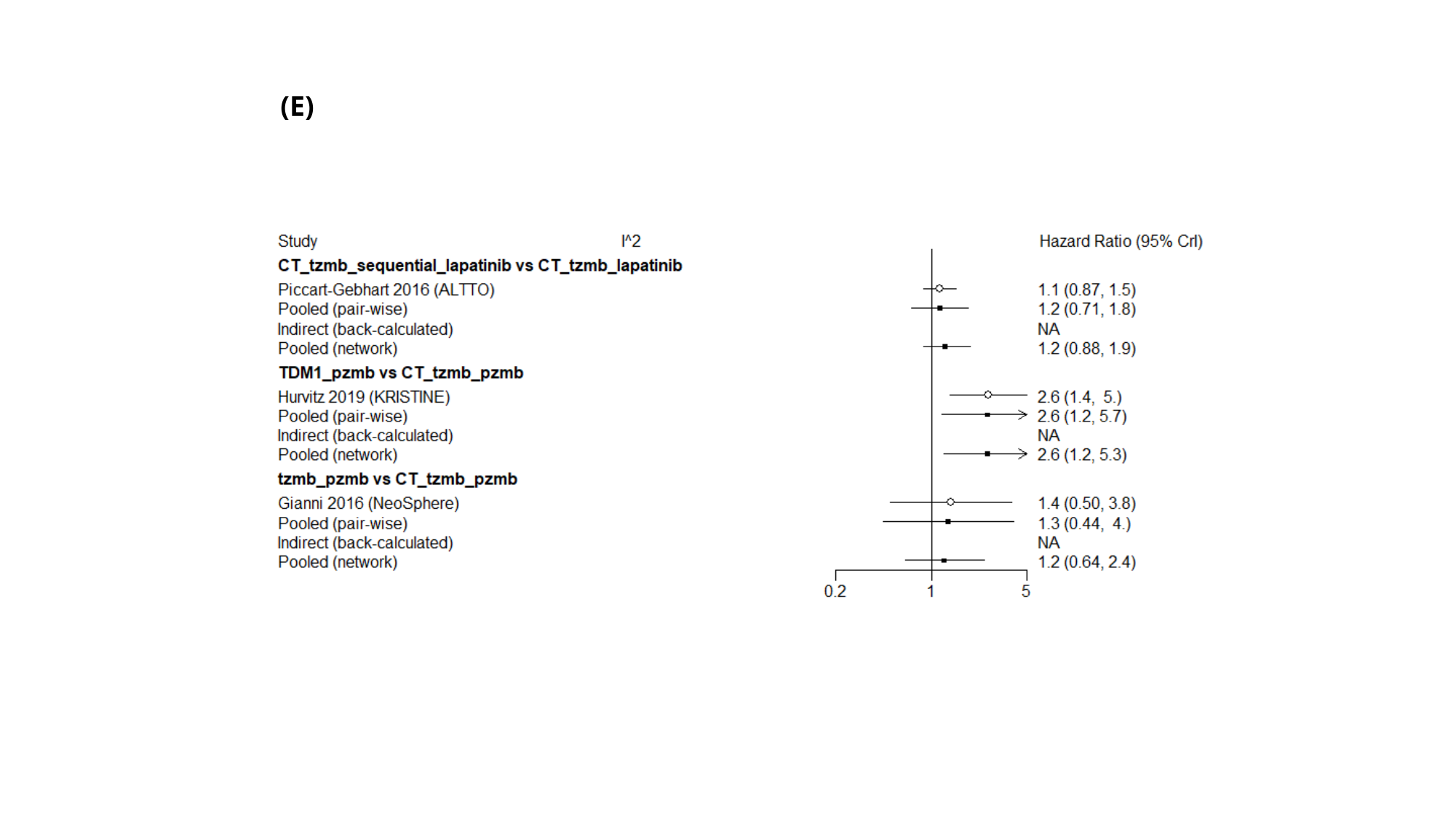

(E)

## Slide 6
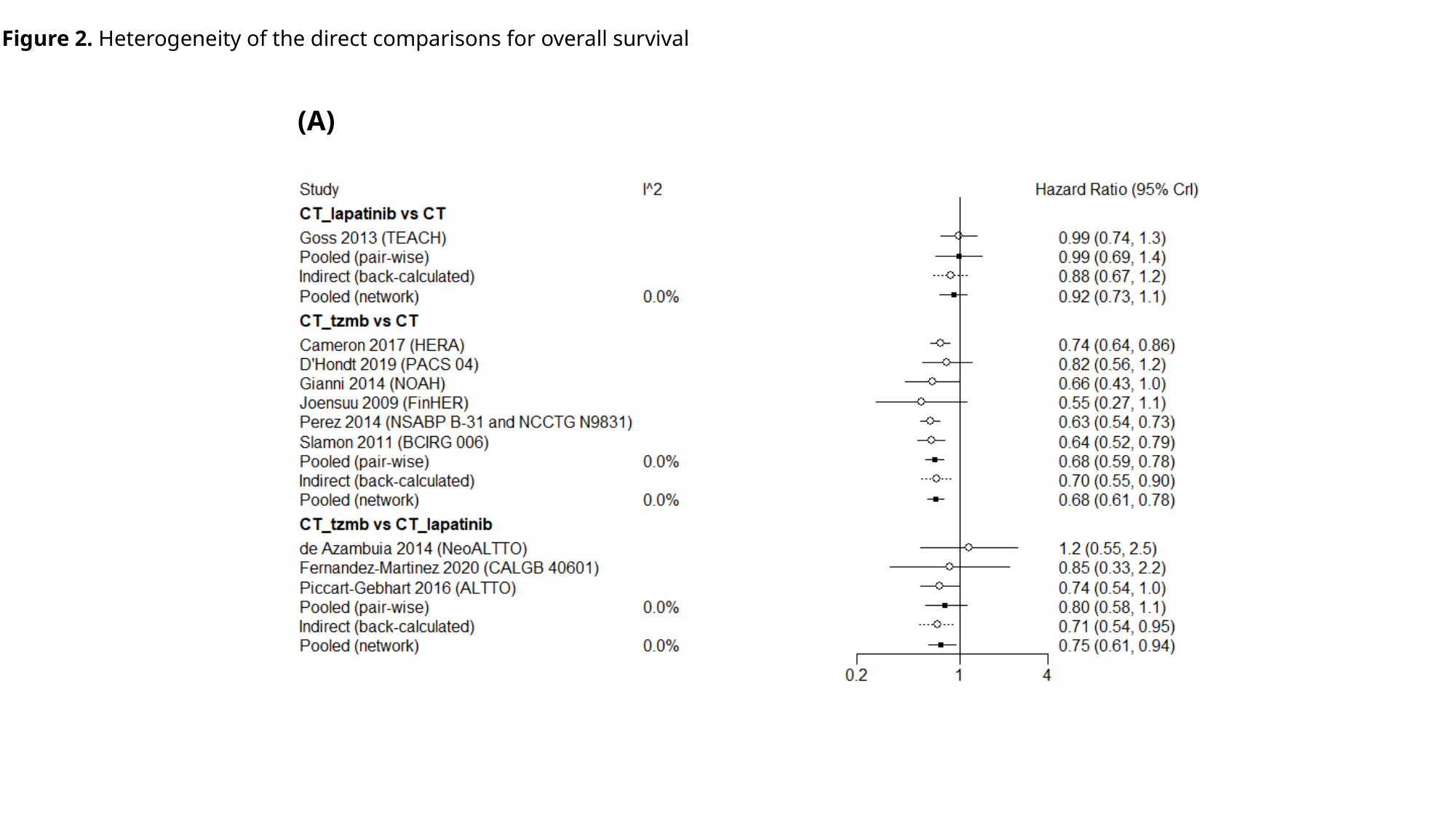

Figure 2. Heterogeneity of the direct comparisons for overall survival
(A)

## Slide 7
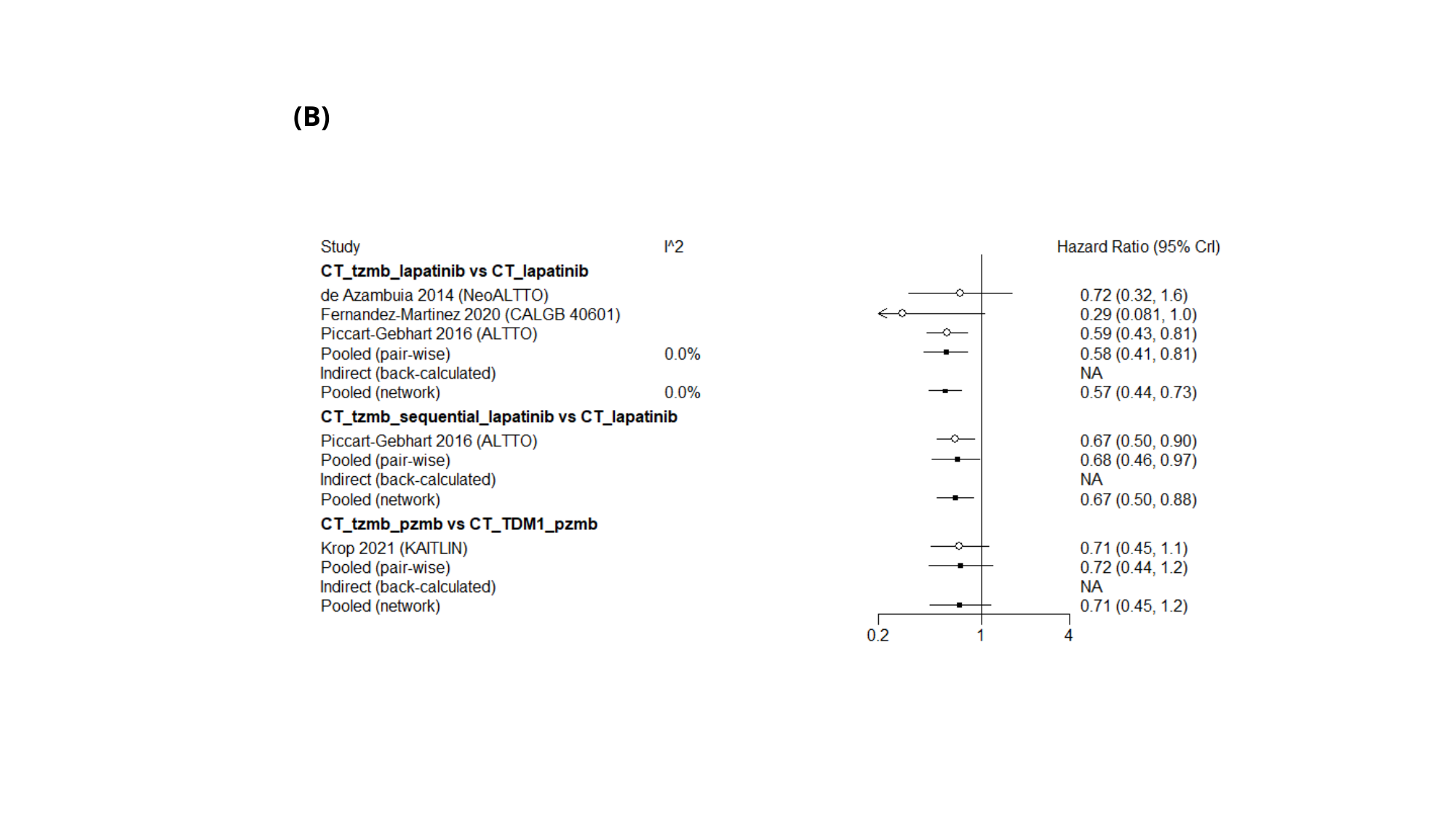

(B)

## Slide 8
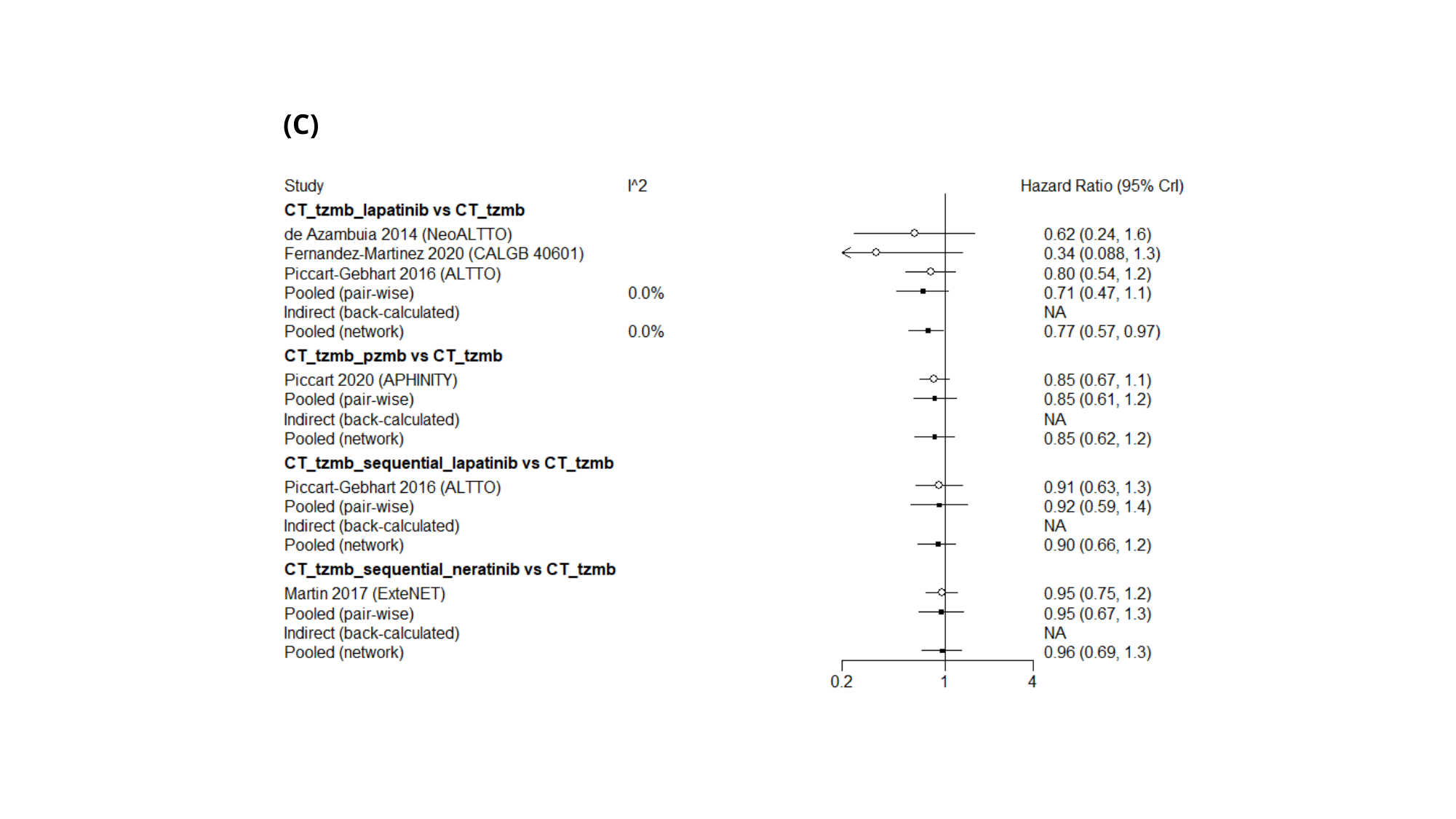

(C)

## Slide 9
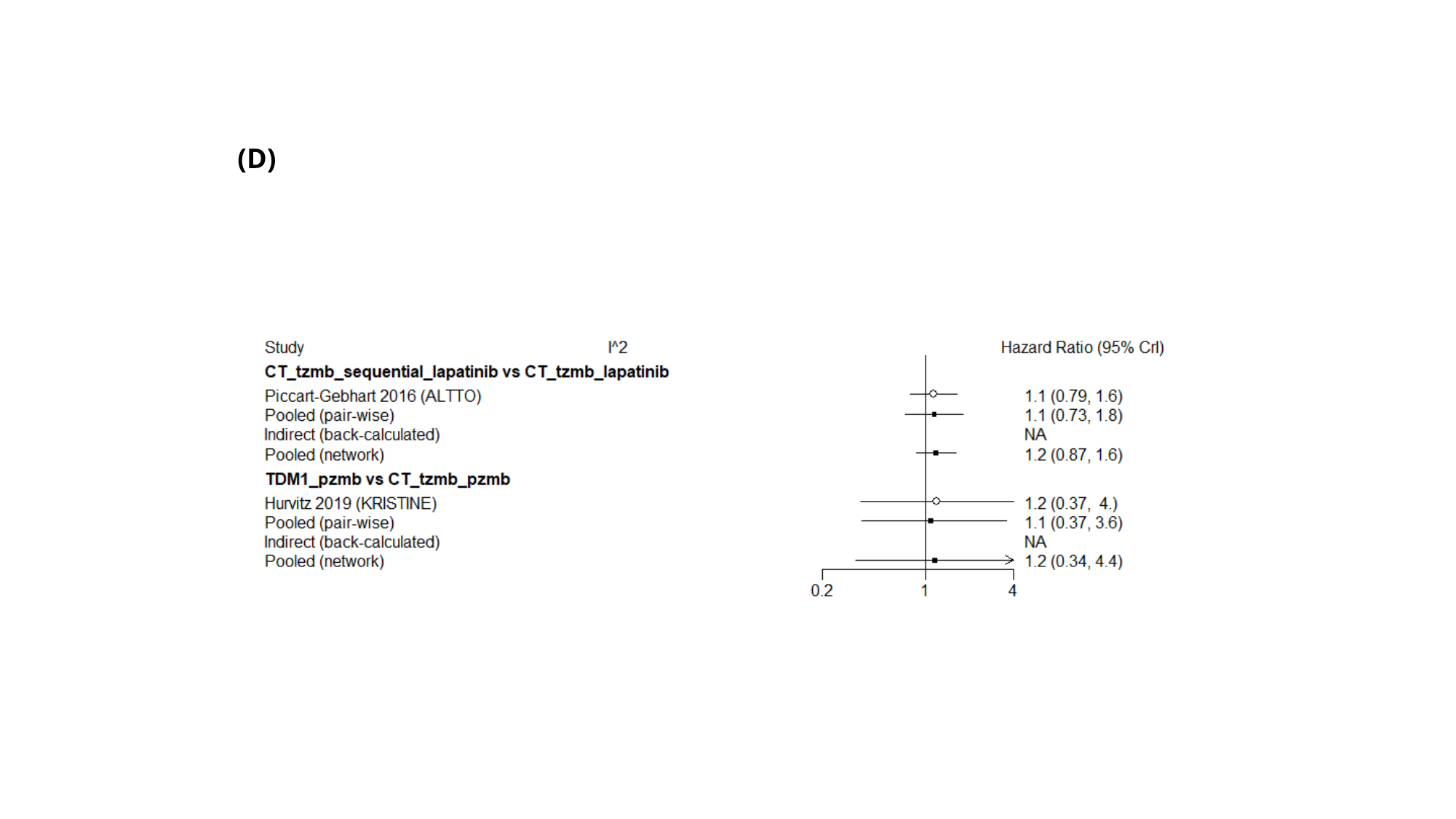

(D)
