# Supplementary material for: Determining the Optimal (Neo)Adjuvant Regimen for Human Epidermal Growth Factor Receptor 2-Positive Breast Cancer Regarding Survival Outcome: A Network Meta-Analysis
Source: Front Immunol. 2022 Jun 30;13:919369. doi: 10.3389/fimmu.2022.919369 (PMC9279606; doi:10.3389/fimmu.2022.919369)
Supplement: Supplementary file 1 [file DataSheet_1.zip › Supplementary Materials/Supplementary Material 4.pptx]

## Slide 1
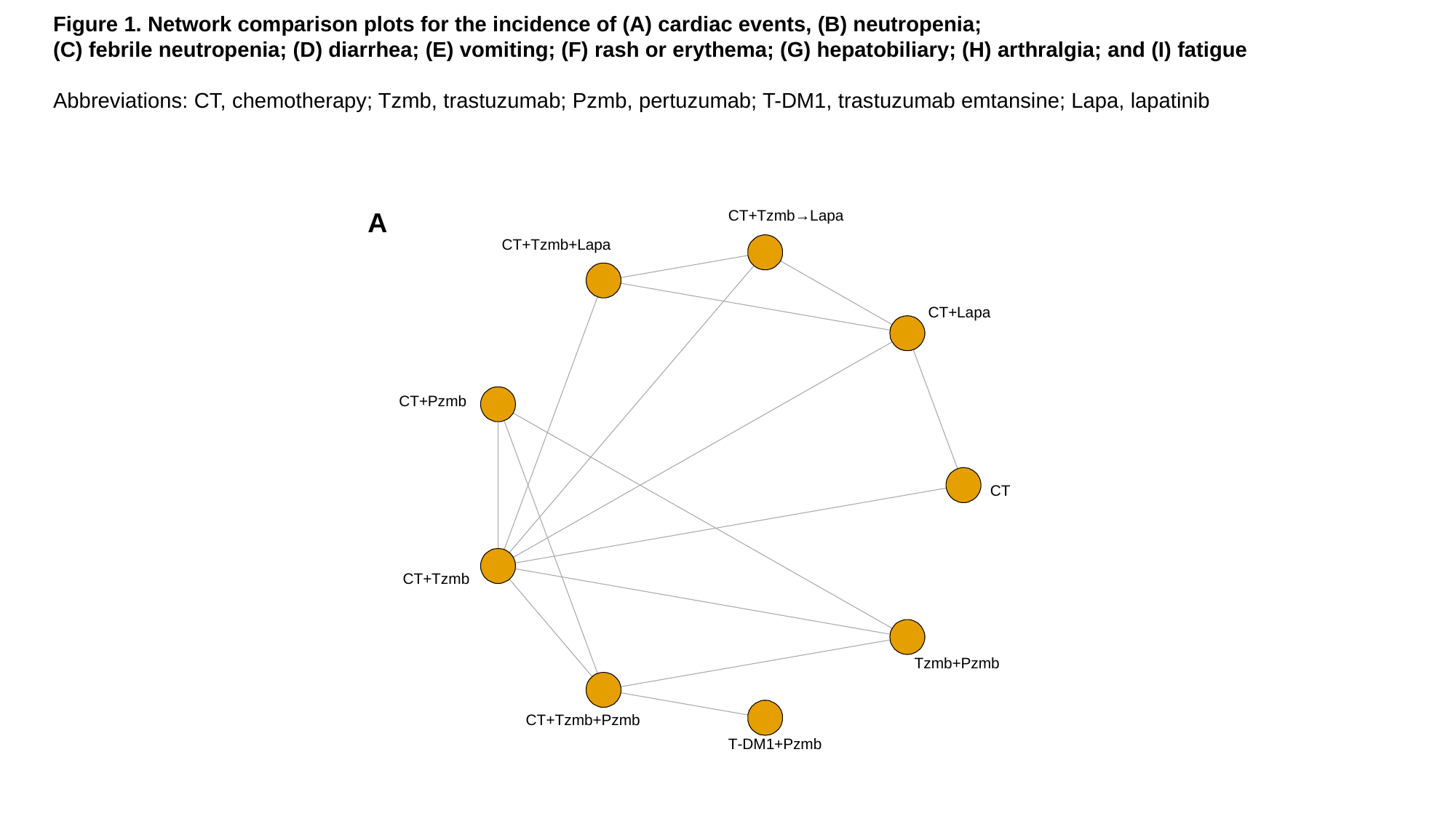

Figure 1. Network comparison plots for the incidence of (A) cardiac events, (B) neutropenia;
(C) febrile neutropenia; (D) diarrhea; (E) vomiting; (F) rash or erythema; (G) hepatobiliary; (H) arthralgia; and (I) fatigue
Abbreviations: CT, chemotherapy; Tzmb, trastuzumab; Pzmb, pertuzumab; T-DM1, trastuzumab emtansine; Lapa, lapatinib
A
CT+Tzmb→Lapa
CT+Tzmb+Lapa
CT+Lapa
CT+Pzmb
CT
CT+Tzmb
Tzmb+Pzmb
CT+Tzmb+Pzmb
T-DM1+Pzmb

## Slide 2
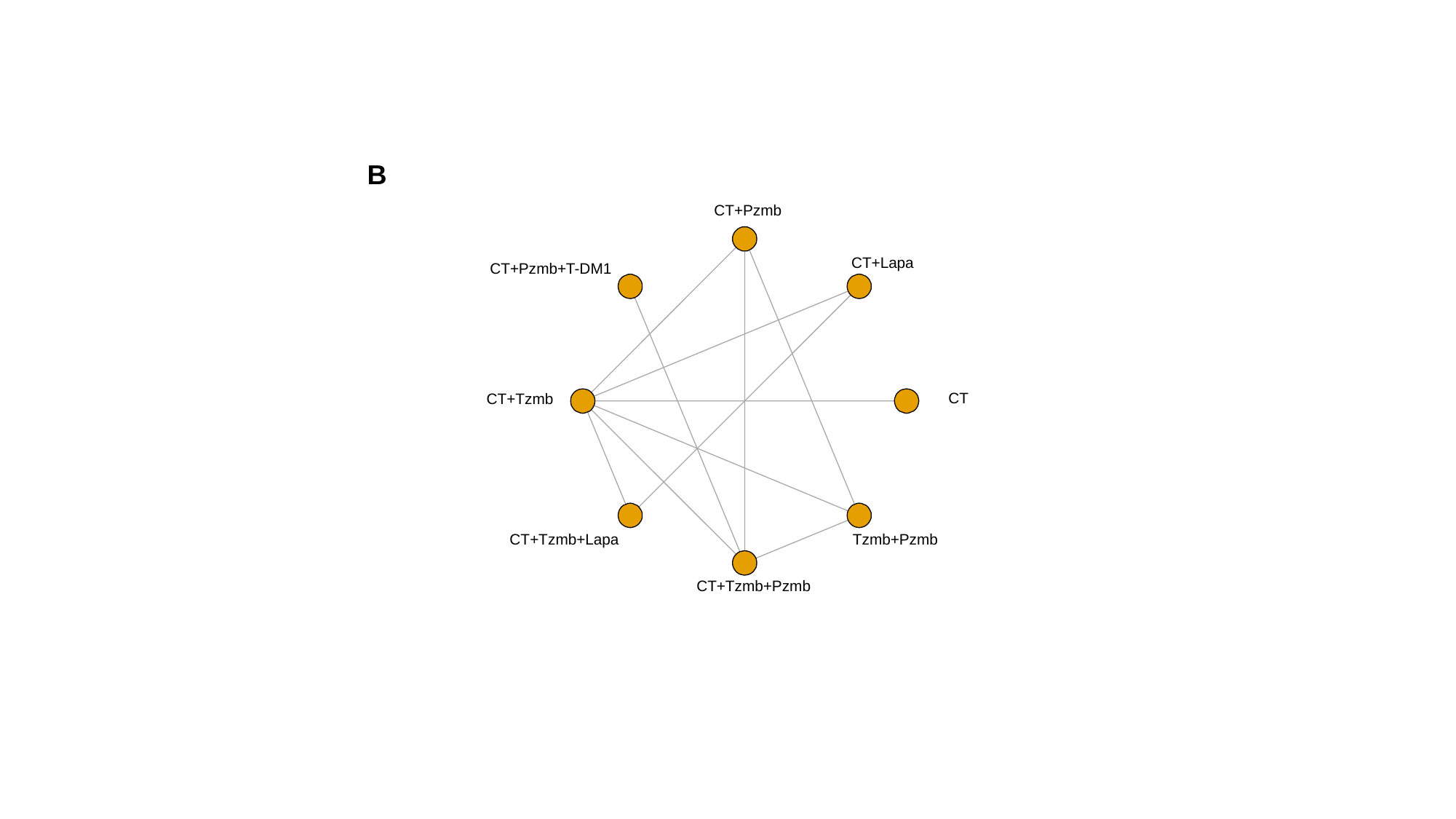

B
CT+Pzmb
CT+Lapa
CT+Pzmb+T-DM1
CT
CT+Tzmb
Tzmb+Pzmb
CT+Tzmb+Lapa
CT+Tzmb+Pzmb

## Slide 3
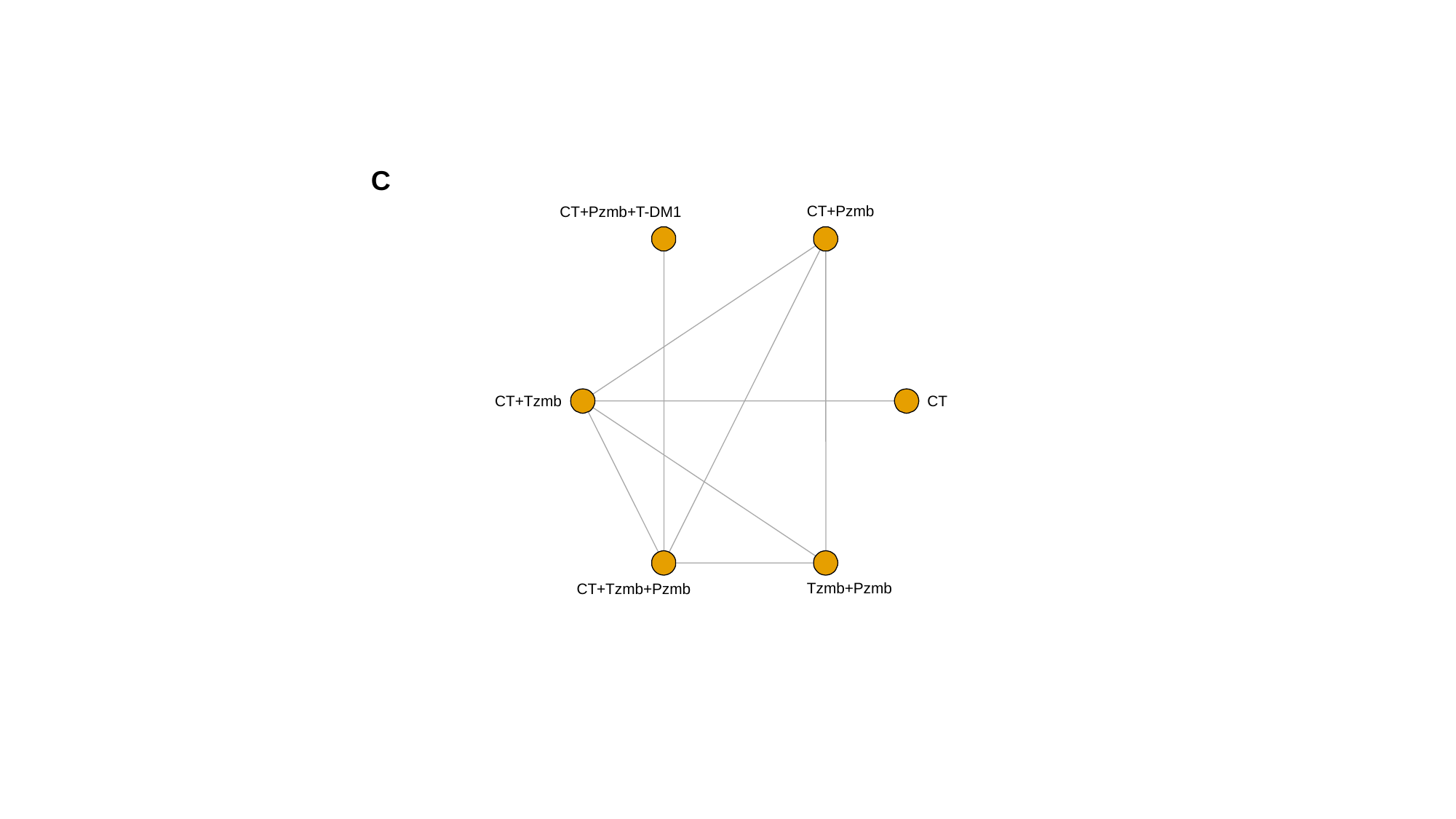

C
CT+Pzmb
CT+Pzmb+T-DM1
CT+Tzmb
CT
Tzmb+Pzmb
CT+Tzmb+Pzmb

## Slide 4
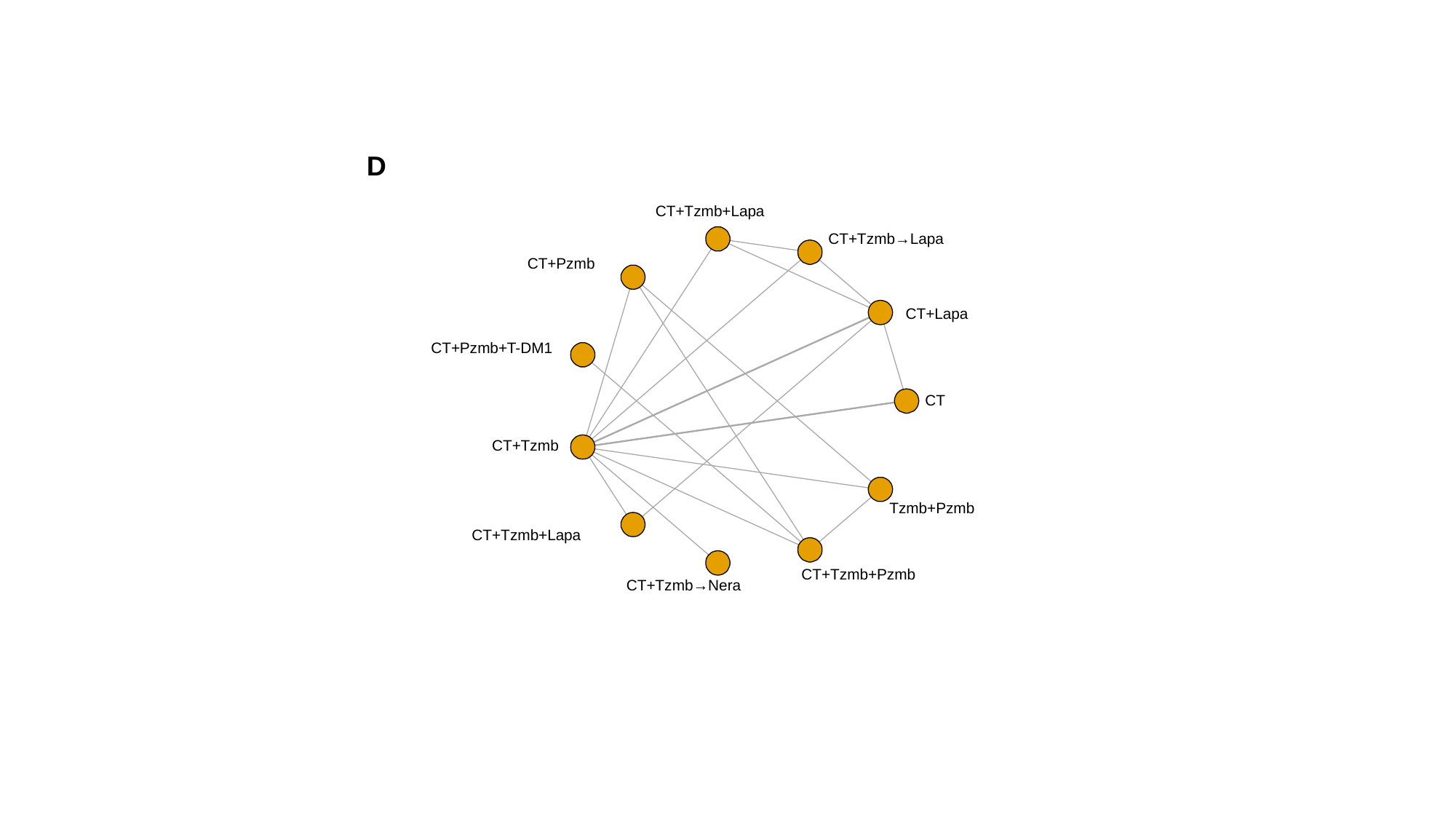

D
CT+Tzmb+Lapa
CT+Tzmb→Lapa
CT+Pzmb
CT+Lapa
CT+Pzmb+T-DM1
CT
CT+Tzmb
Tzmb+Pzmb
CT+Tzmb+Lapa
CT+Tzmb+Pzmb
CT+Tzmb→Nera

## Slide 5
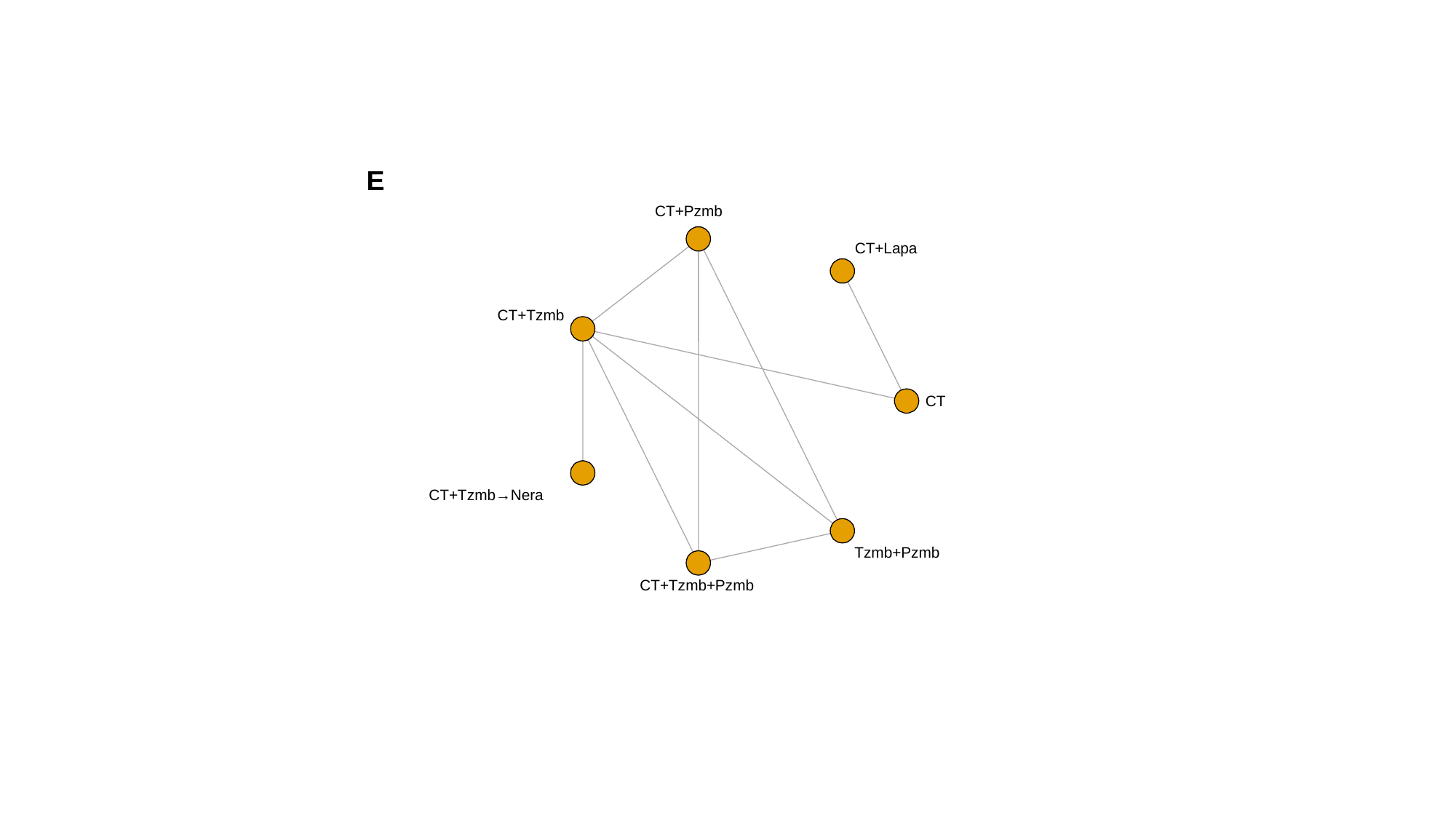

E
CT+Pzmb
CT+Lapa
CT+Tzmb
CT
CT+Tzmb→Nera
Tzmb+Pzmb
CT+Tzmb+Pzmb

## Slide 6
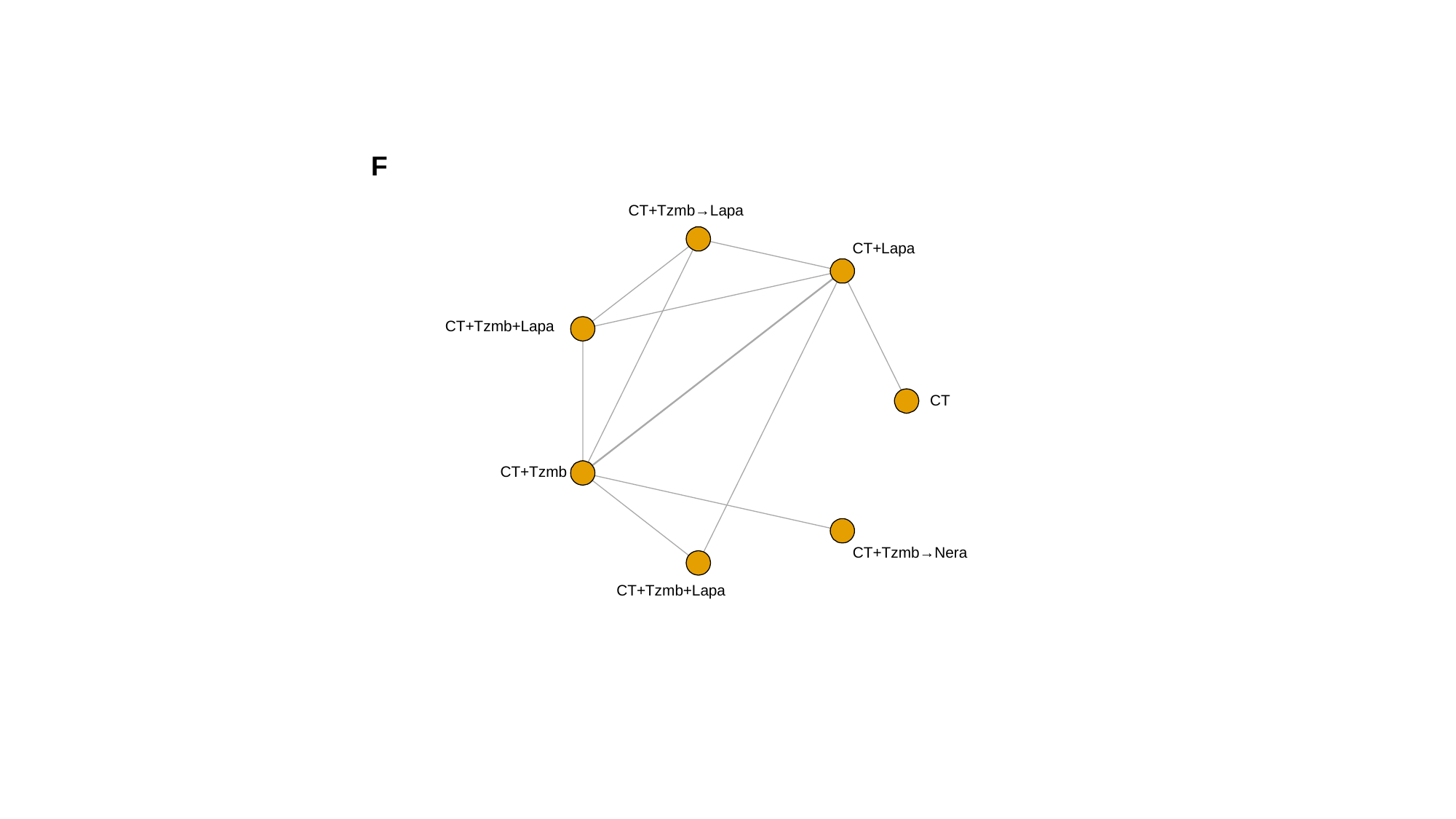

F
CT+Tzmb→Lapa
CT+Lapa
CT+Tzmb+Lapa
CT
CT+Tzmb
CT+Tzmb→Nera
CT+Tzmb+Lapa

## Slide 7
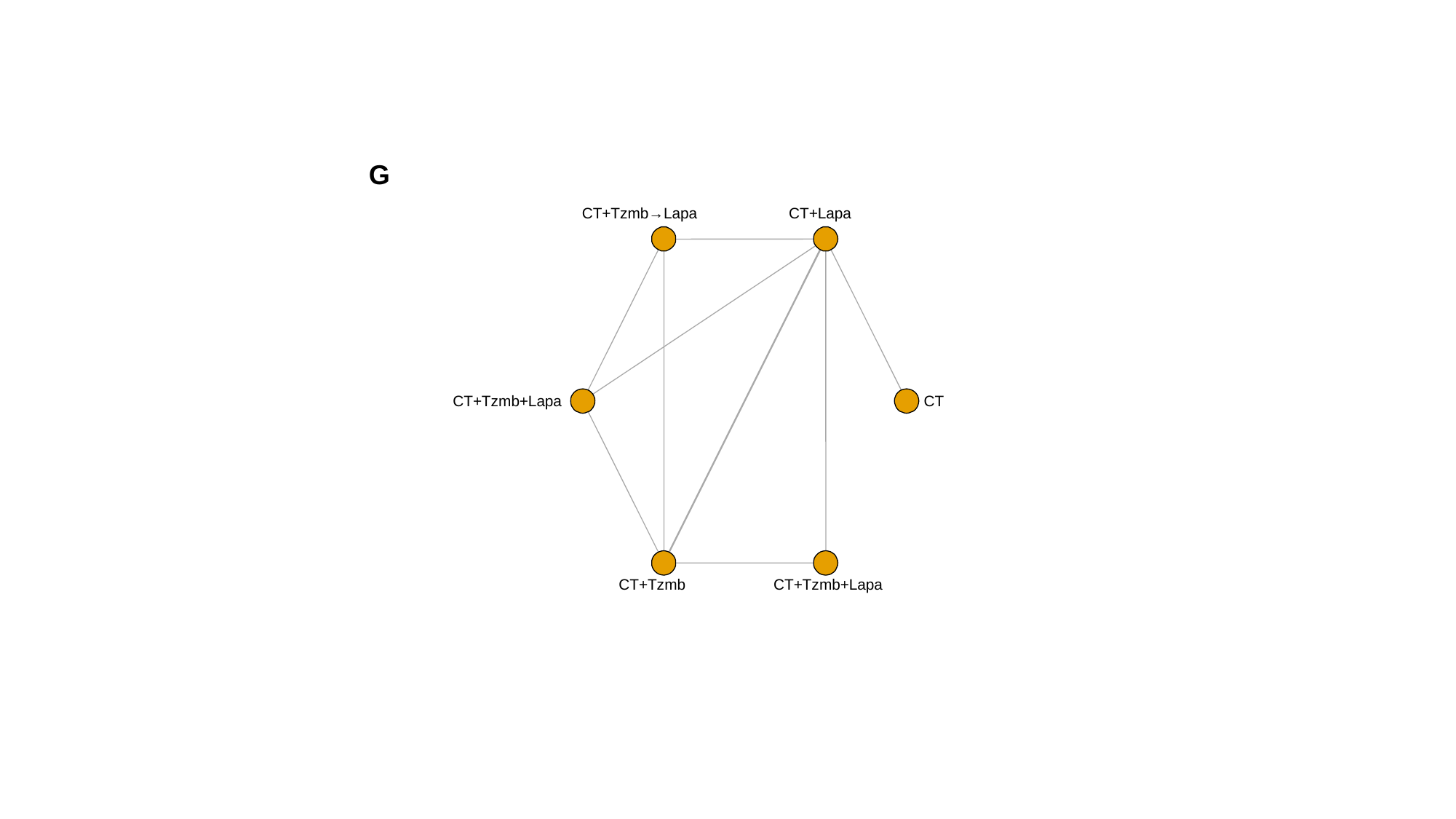

G
CT+Tzmb→Lapa
CT+Lapa
CT
CT+Tzmb+Lapa
CT+Tzmb
CT+Tzmb+Lapa

## Slide 8
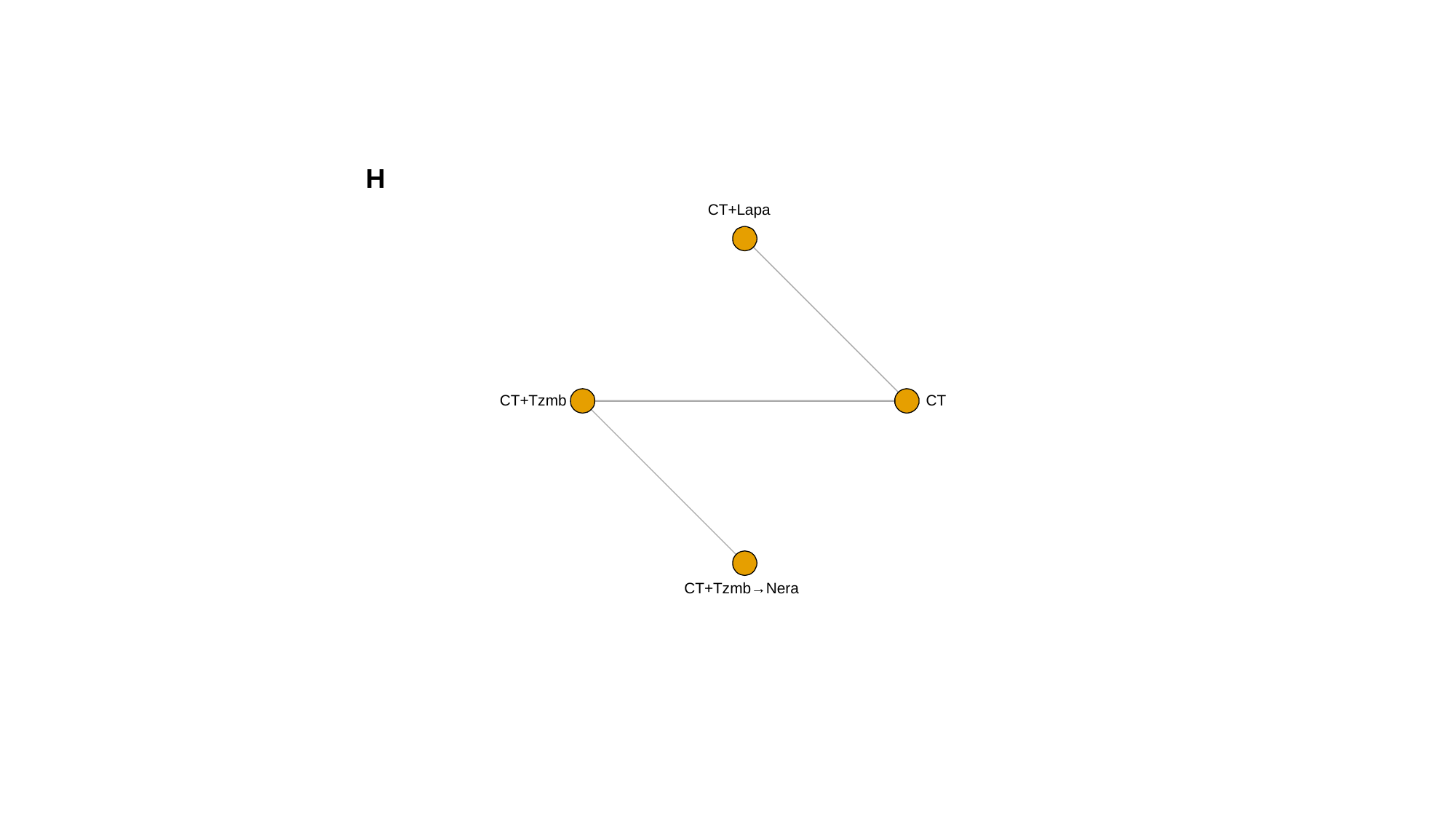

H
CT+Lapa
CT+Tzmb
CT
CT+Tzmb→Nera

## Slide 9
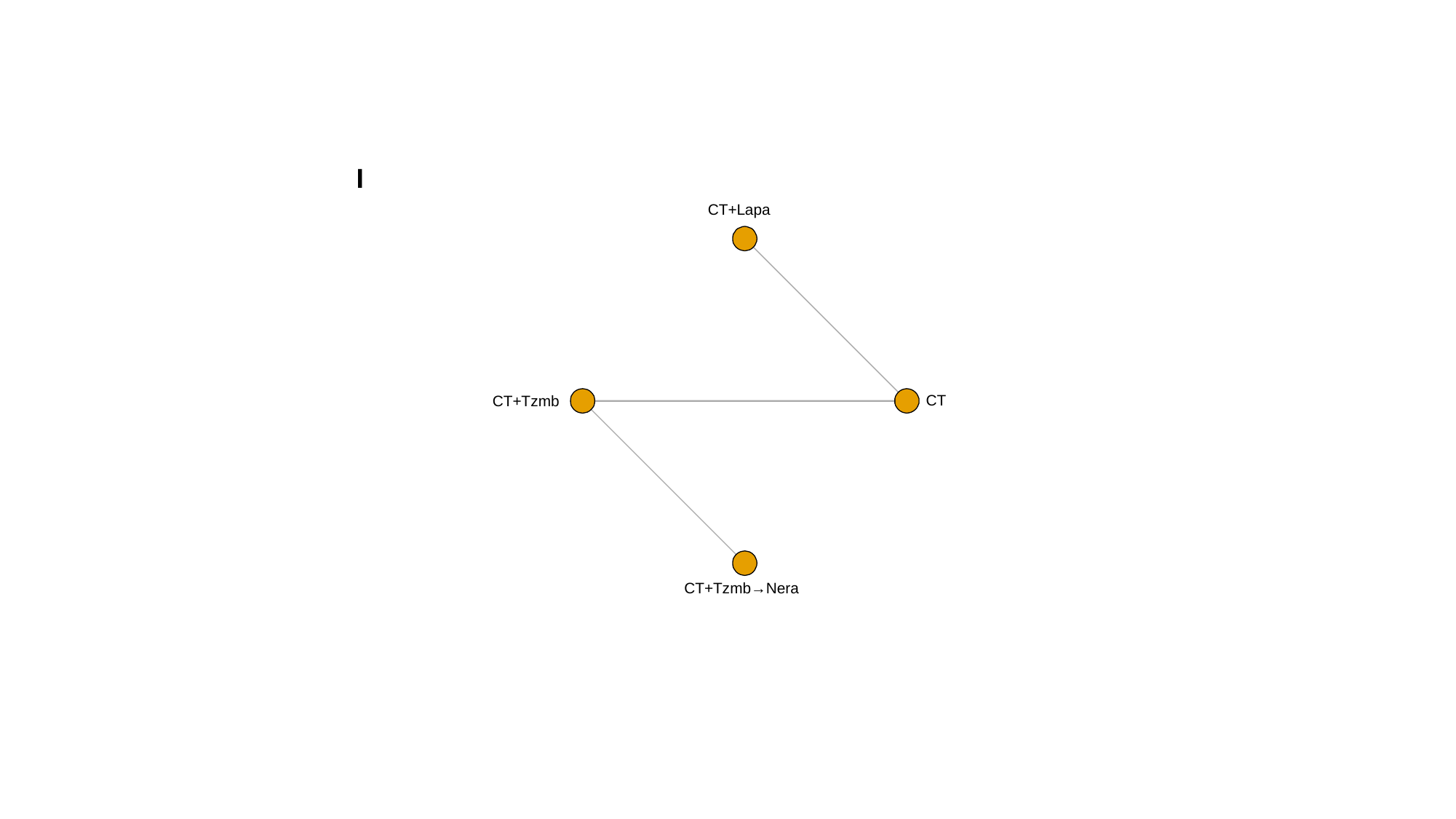

I
CT+Lapa
CT
CT+Tzmb
CT+Tzmb→Nera
