# Supplementary material for: Determining the Optimal (Neo)Adjuvant Regimen for Human Epidermal Growth Factor Receptor 2-Positive Breast Cancer Regarding Survival Outcome: A Network Meta-Analysis
Source: Front Immunol. 2022 Jun 30;13:919369. doi: 10.3389/fimmu.2022.919369 (PMC9279606; doi:10.3389/fimmu.2022.919369)
Supplement: Supplementary file 1 [file DataSheet_1.zip › Supplementary Materials/Supplementary Material 5.pptx]

## Slide 1
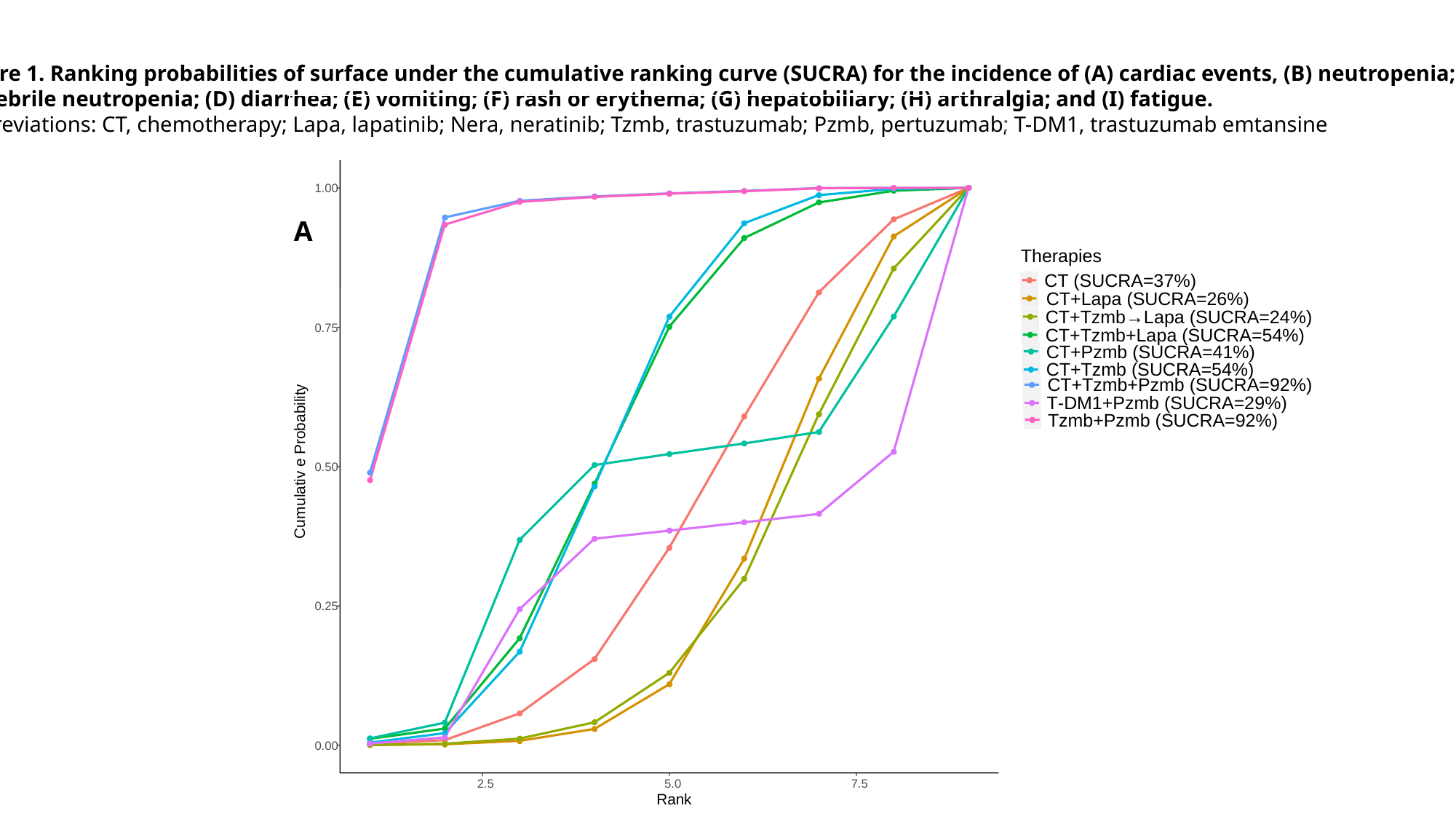

Figure 1. Ranking probabilities of surface under the cumulative ranking curve (SUCRA) for the incidence of (A) cardiac events, (B) neutropenia;
(C) febrile neutropenia; (D) diarrhea; (E) vomiting; (F) rash or erythema; (G) hepatobiliary; (H) arthralgia; and (I) fatigue.
Abbreviations: CT, chemotherapy; Lapa, lapatinib; Nera, neratinib; Tzmb, trastuzumab; Pzmb, pertuzumab; T-DM1, trastuzumab emtansine
1.00
A
Therapies
CT (SUCRA=37%)
CT+Lapa (SUCRA=26%)
CT+Tzmb→Lapa (SUCRA=24%)
0.75
CT+Tzmb+Lapa (SUCRA=54%)
CT+Pzmb (SUCRA=41%)
CT+Tzmb (SUCRA=54%)
CT+Tzmb+Pzmb (SUCRA=92%)
T-DM1+Pzmb (SUCRA=29%)
Tzmb+Pzmb (SUCRA=92%)
e Probability
0.50
v
ulati
m
Cu
0.25
0.00
2.5
5.0
7.5
Rank

## Slide 2
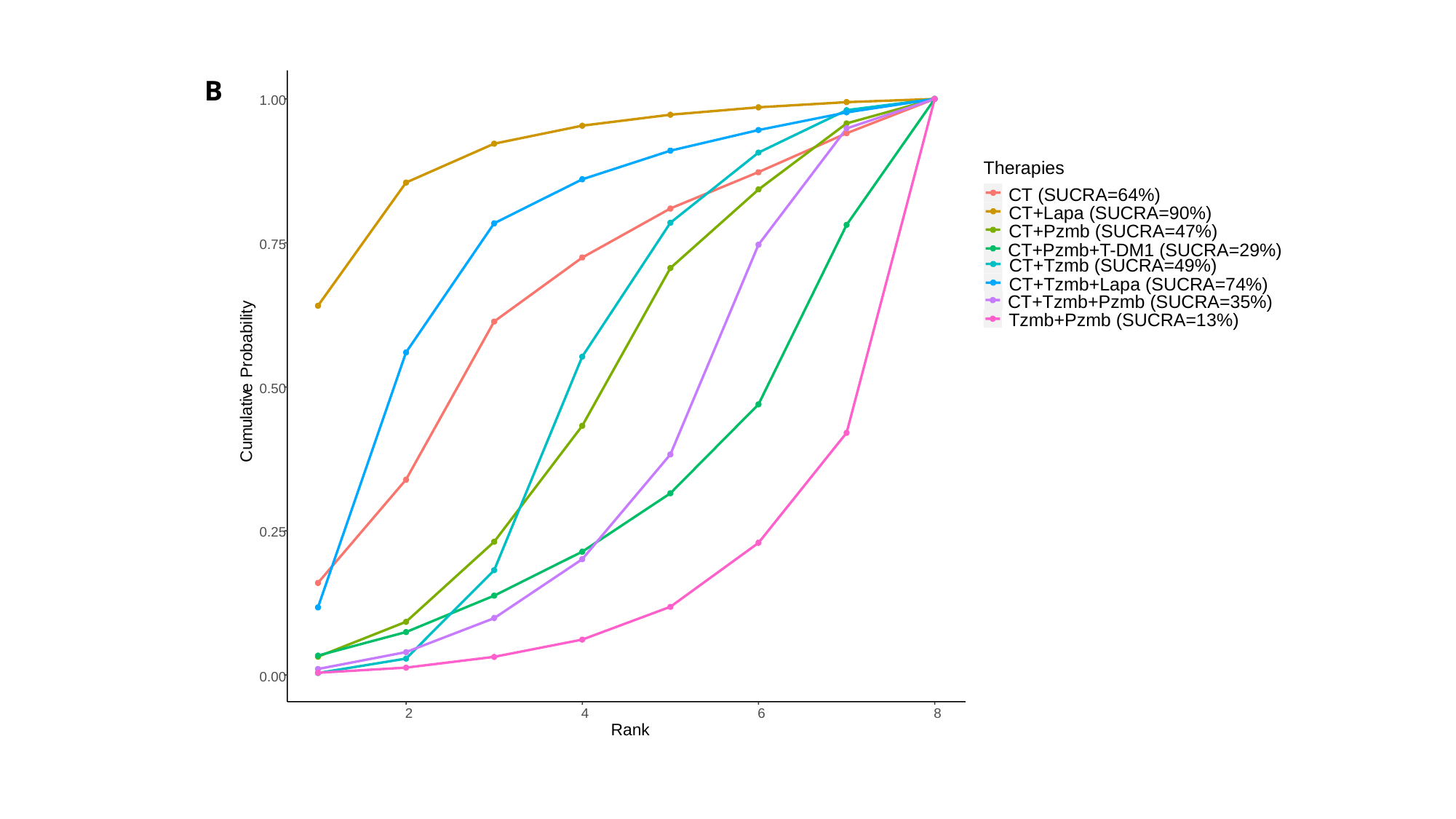

B
1.00
Therapies
CT (SUCRA=64%)
CT+Lapa (SUCRA=90%)
CT+Pzmb (SUCRA=47%)
0.75
CT+Pzmb+T-DM1 (SUCRA=29%)
CT+Tzmb (SUCRA=49%)
CT+Tzmb+Lapa (SUCRA=74%)
CT+Tzmb+Pzmb (SUCRA=35%)
Tzmb+Pzmb (SUCRA=13%)
e Probability
0.50
v
ulati
m
Cu
0.25
0.00
2
4
6
8
Rank

## Slide 3
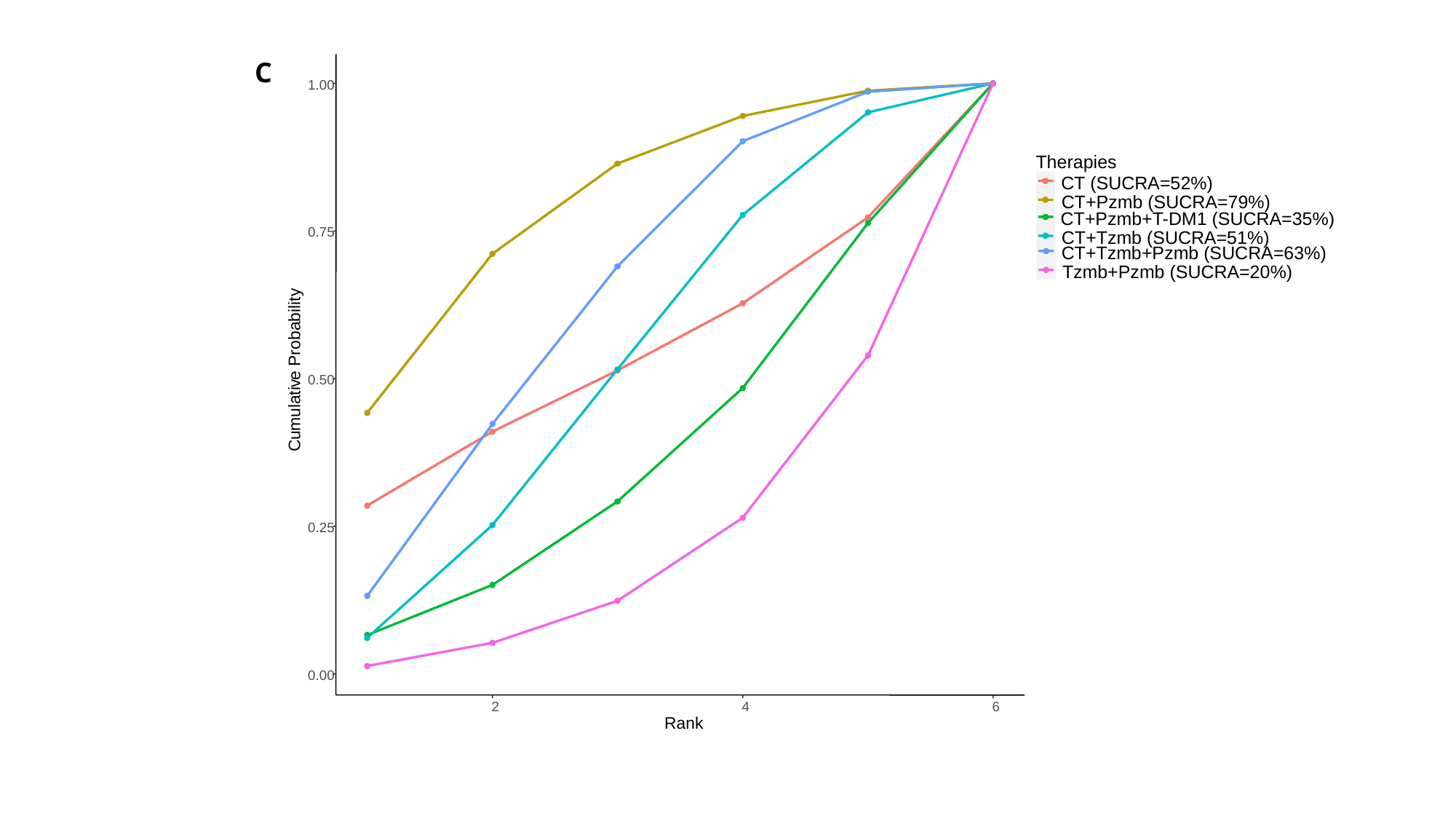

C
1.00
Therapies
CT (SUCRA=52%)
CT+Pzmb (SUCRA=79%)
CT+Pzmb+T-DM1 (SUCRA=35%)
0.75
CT+Tzmb (SUCRA=51%)
CT+Tzmb+Pzmb (SUCRA=63%)
Tzmb+Pzmb (SUCRA=20%)
e Probability
0.50
v
ulati
m
Cu
0.25
0.00
2
4
6
Rank

## Slide 4
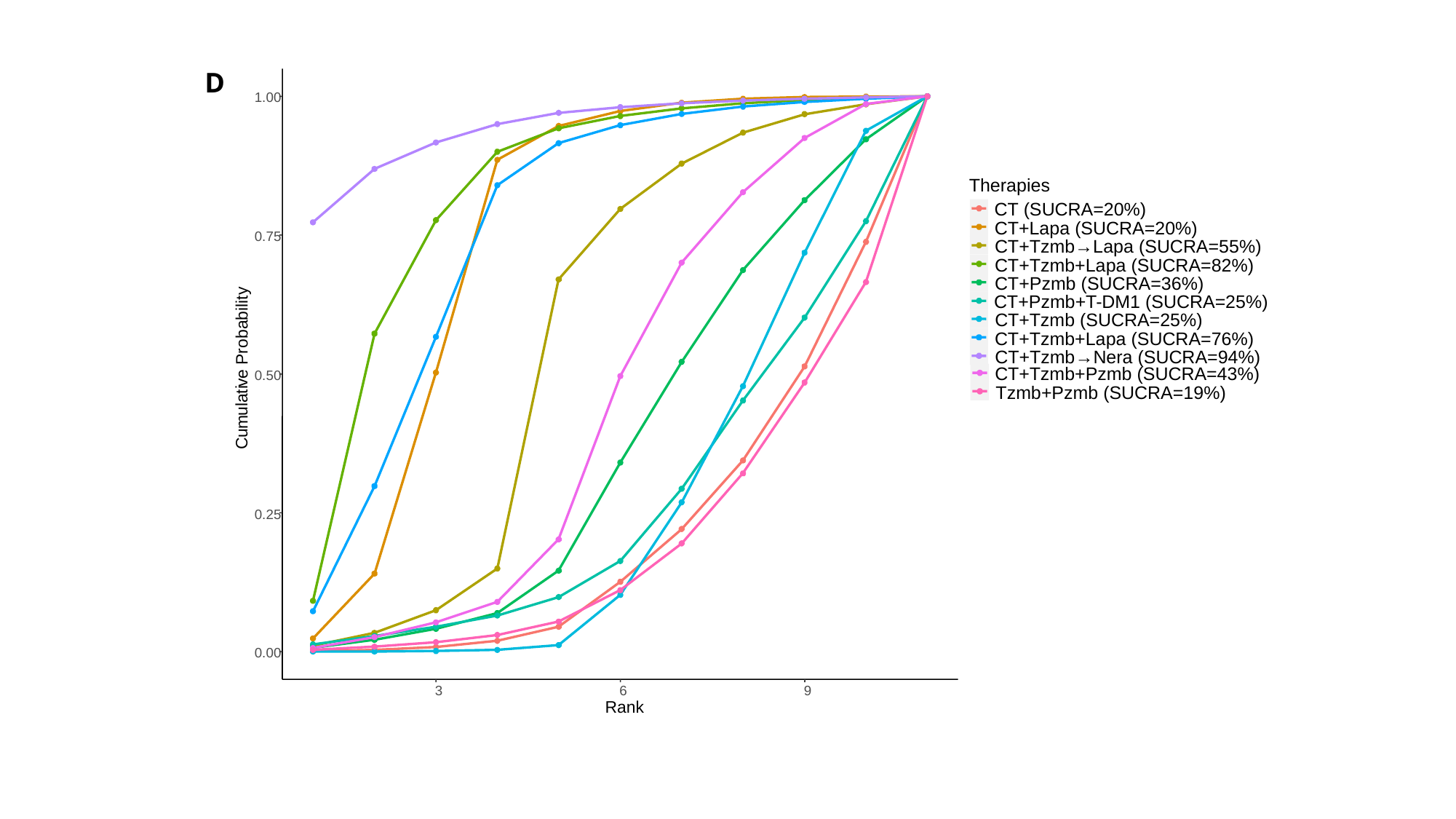

D
1.00
Therapies
CT (SUCRA=20%)
CT+Lapa (SUCRA=20%)
0.75
CT+Tzmb→Lapa (SUCRA=55%)
CT+Tzmb+Lapa (SUCRA=82%)
CT+Pzmb (SUCRA=36%)
CT+Pzmb+T-DM1 (SUCRA=25%)
CT+Tzmb (SUCRA=25%)
e Probability
CT+Tzmb+Lapa (SUCRA=76%)
CT+Tzmb→Nera (SUCRA=94%)
CT+Tzmb+Pzmb (SUCRA=43%)
0.50
v
Tzmb+Pzmb (SUCRA=19%)
ulati
m
Cu
0.25
0.00
3
6
9
Rank

## Slide 5
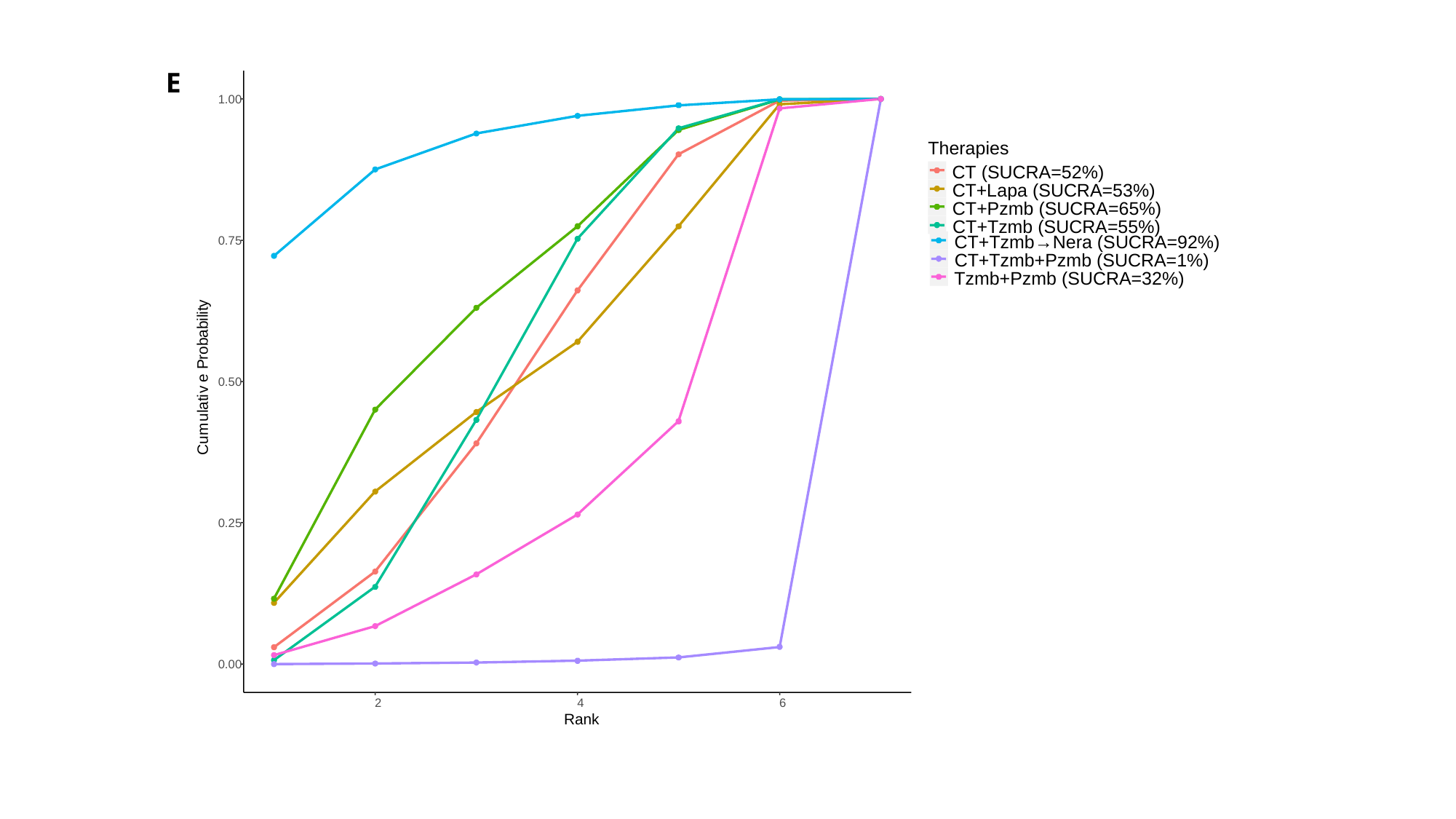

E
1.00
Therapies
CT (SUCRA=52%)
CT+Lapa (SUCRA=53%)
CT+Pzmb (SUCRA=65%)
CT+Tzmb (SUCRA=55%)
CT+Tzmb→Nera (SUCRA=92%)
0.75
CT+Tzmb+Pzmb (SUCRA=1%)
Tzmb+Pzmb (SUCRA=32%)
e Probability
0.50
v
ulati
m
Cu
0.25
0.00
2
4
6
Rank

## Slide 6
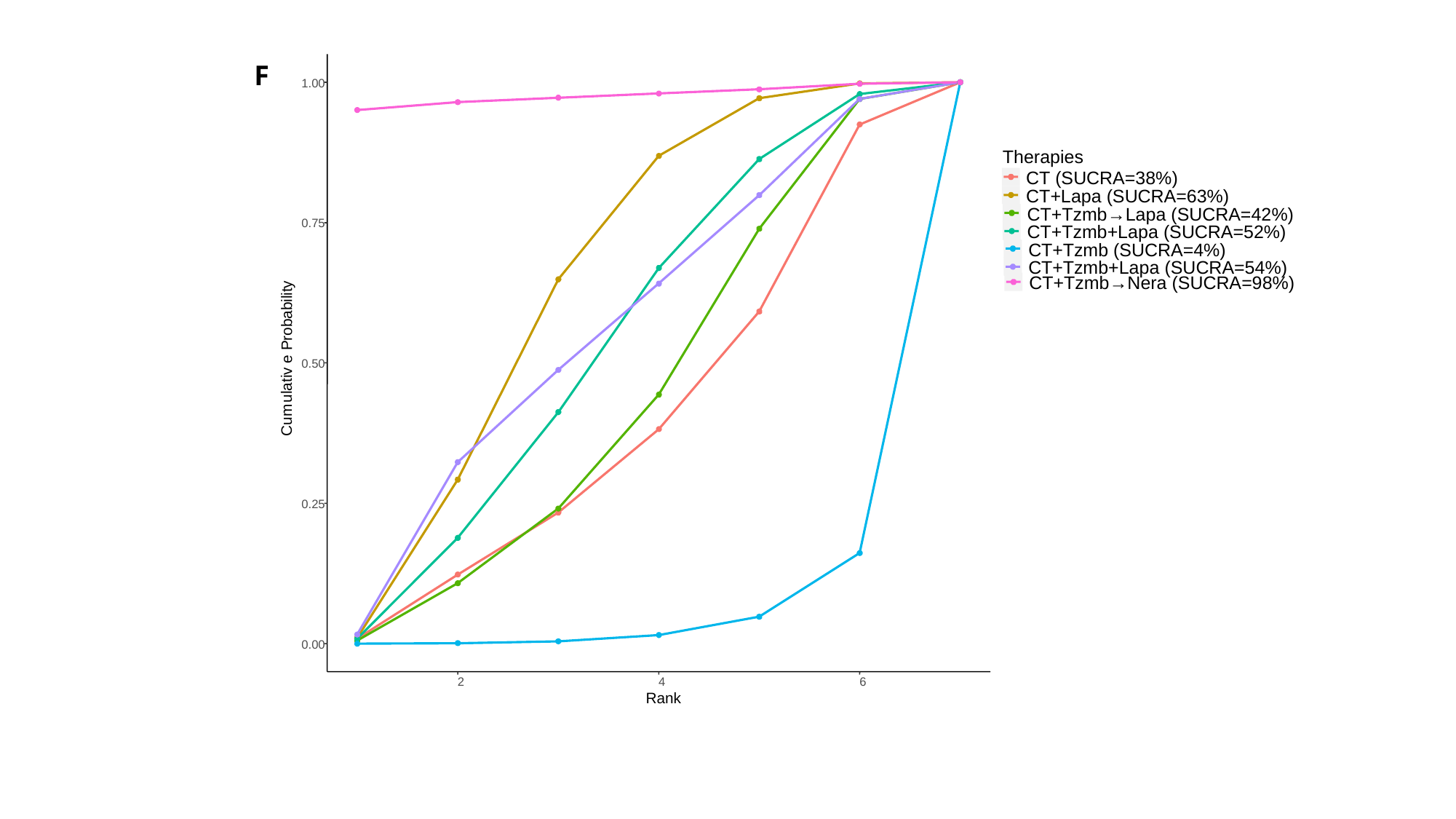

F
1.00
Therapies
CT (SUCRA=38%)
CT+Lapa (SUCRA=63%)
CT+Tzmb→Lapa (SUCRA=42%)
0.75
CT+Tzmb+Lapa (SUCRA=52%)
CT+Tzmb (SUCRA=4%)
CT+Tzmb+Lapa (SUCRA=54%)
CT+Tzmb→Nera (SUCRA=98%)
e Probability
0.50
v
ulati
m
Cu
0.25
0.00
2
4
6
Rank

## Slide 7
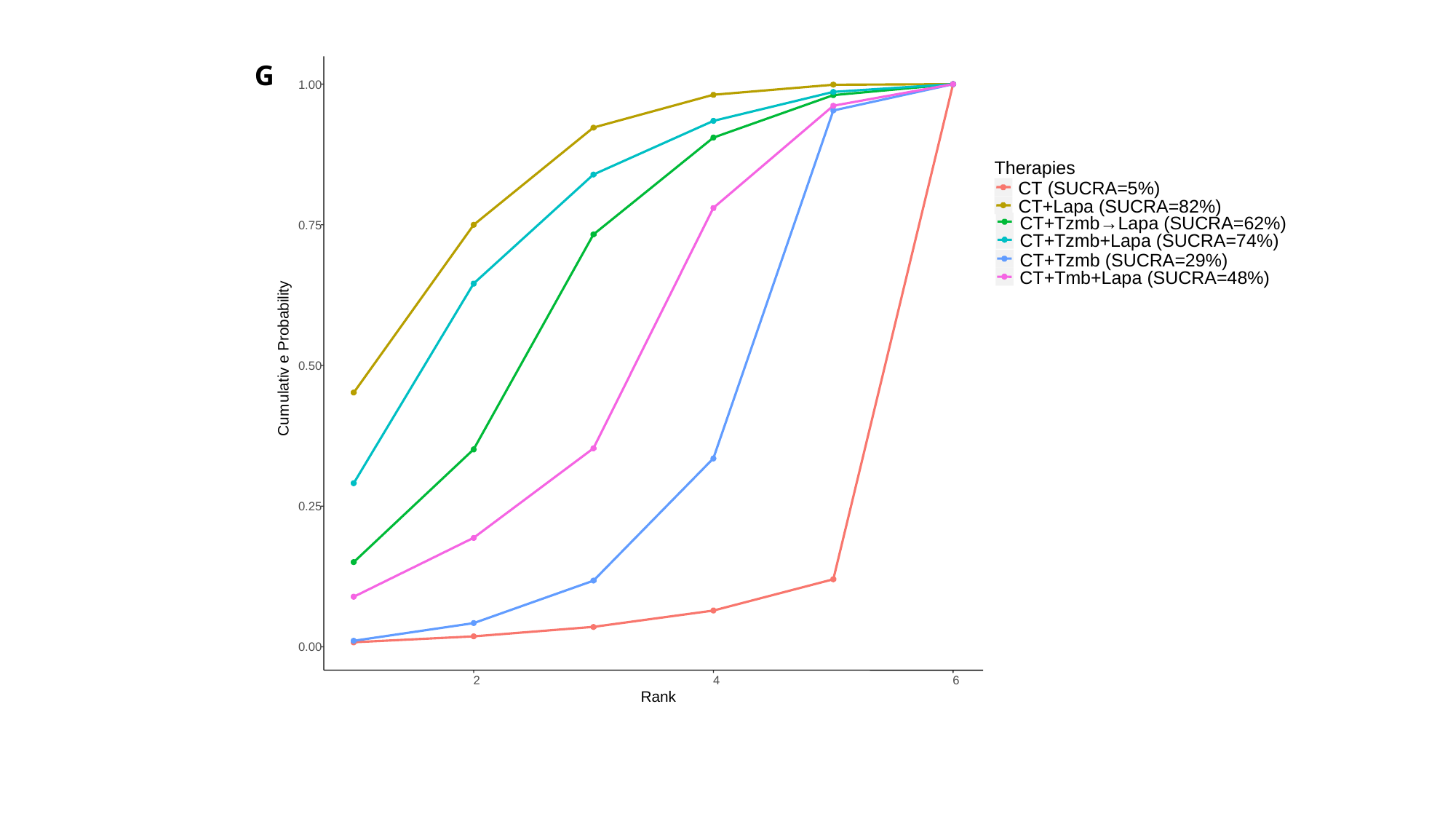

G
1.00
Therapies
CT (SUCRA=5%)
CT+Lapa (SUCRA=82%)
CT+Tzmb→Lapa (SUCRA=62%)
0.75
CT+Tzmb+Lapa (SUCRA=74%)
CT+Tzmb (SUCRA=29%)
CT+Tmb+Lapa (SUCRA=48%)
e Probability
0.50
v
ulati
m
Cu
0.25
0.00
2
4
6
Rank

## Slide 8
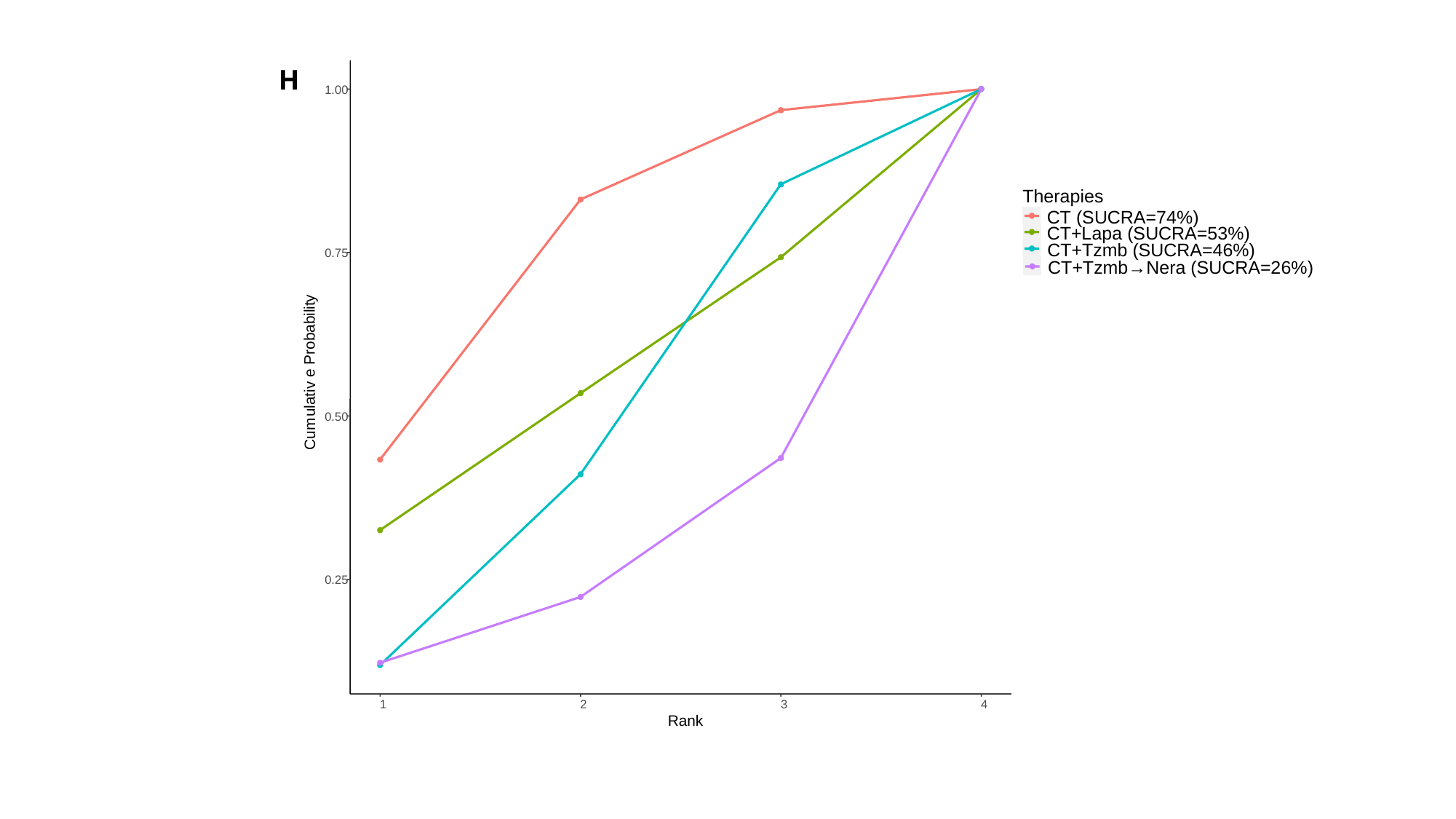

H
1.00
Therapies
CT (SUCRA=74%)
CT+Lapa (SUCRA=53%)
CT+Tzmb (SUCRA=46%)
0.75
CT+Tzmb→Nera (SUCRA=26%)
e Probability
v
ulati
0.50
m
Cu
0.25
1
2
3
4
Rank

## Slide 9
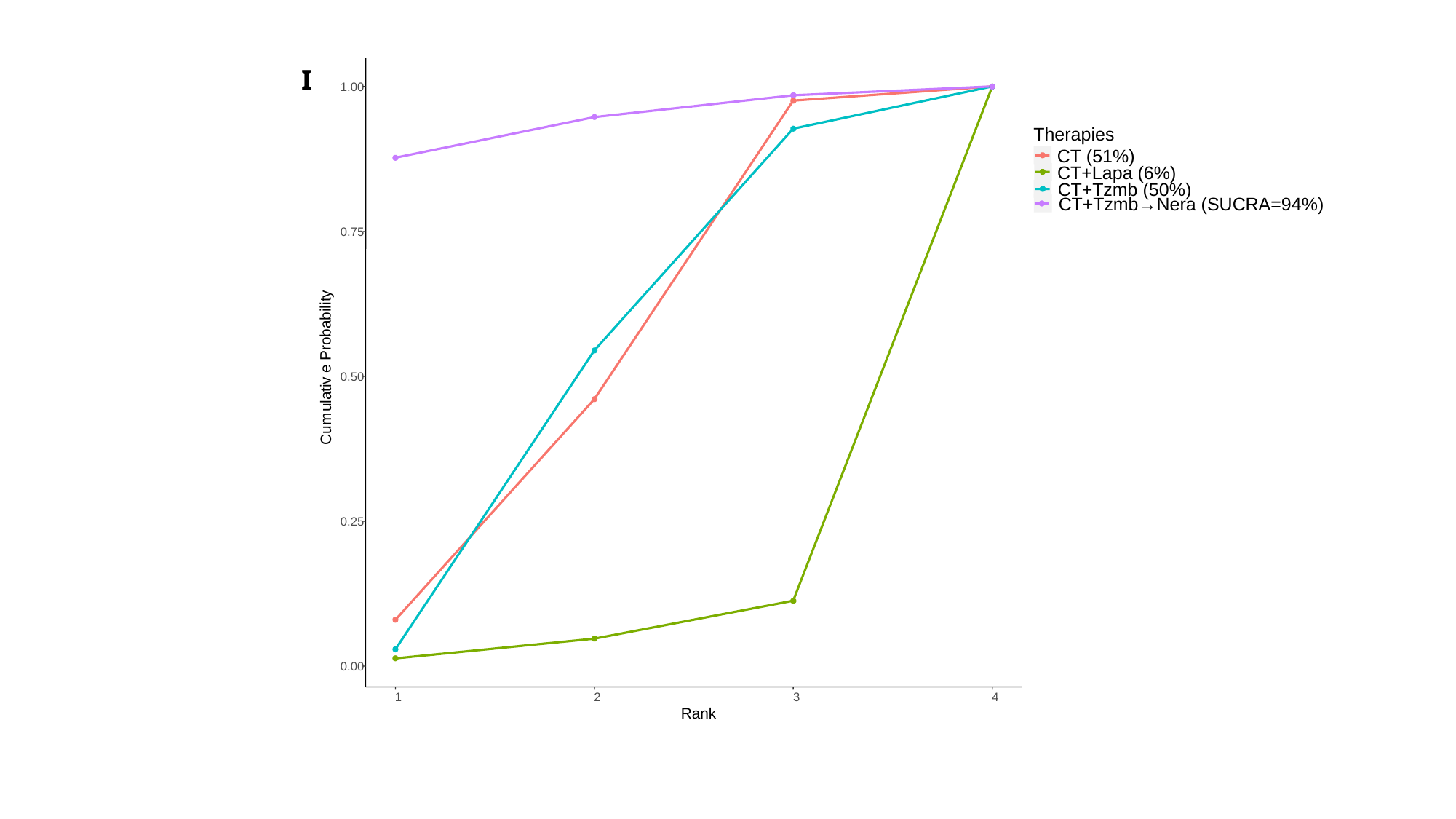

I
1.00
Therapies
CT (51%)
CT+Lapa (6%)
CT+Tzmb (50%)
CT+Tzmb→Nera (SUCRA=94%)
0.75
e Probability
v
0.50
ulati
m
Cu
0.25
0.00
1
2
3
4
Rank
